# The PXDLS linear motif regulates circadian rhythmicity through protein–protein interactions

**DOI:** 10.1093/nar/gku873

**Published:** 2014-09-26

**Authors:** Moran Shalev, Rona Aviram, Yaarit Adamovich, Judith Kraut-Cohen, Tal Shamia, Shifra Ben-Dor, Marina Golik, Gad Asher

**Affiliations:** 1 Department of Biological Chemistry, Weizmann Institute of Science, Rehovot 76100, Israel; 2 Biological Services, Weizmann Institute of Science, Rehovot 76100, Israel

## Abstract

The circadian core clock circuitry relies on interlocked transcription-translation feedback loops that largely count on multiple protein interactions. The molecular mechanisms implicated in the assembly of these protein complexes are relatively unknown. Our bioinformatics analysis of short linear motifs, implicated in protein interactions, reveals an enrichment of the Pro-X-Asp-Leu-Ser (PXDLS) motif within circadian transcripts. We show that the PXDLS motif can bind to BMAL1/CLOCK and disrupt circadian oscillations in a cell-autonomous manner. Remarkably, the motif is evolutionary conserved in the core clock protein REV-ERBα, and additional proteins implicated in the clock's function (NRIP1, CBP). In this conjuncture, we uncover a novel cross talk between the two principal core clock feedback loops and show that BMAL/CLOCK and REV-ERBα interact and that the PXDLS motif of REV-ERBα participates in their binding. Furthermore, we demonstrate that the PXDLS motifs of NRIP1 and CBP are involved in circadian rhythmicity. Our findings suggest that the PXDLS motif plays an important role in circadian rhythmicity through regulation of protein interactions within the clock circuitry and that short linear motifs can be employed to modulate circadian oscillations.

## INTRODUCTION

Light-sensitive organisms possess a circadian timekeeping system that serves to coordinate physiology and behavior with the geophysical time. The mammalian circadian timing system consists of a master clock in the brain that responds to light-dark cycles and synchronizes peripheral oscillators, found in almost every cell of the body. Both the master and peripheral clocks share a similar molecular makeup for rhythm generation, and tick in a self-sustained and cell-autonomous manner in cultured cells and living animals (1,2).

The rhythm-generating molecular machinery functions based on intricate interlocked transcription-translation feedback loops that heavily rely on multiple protein interactions (3–5). The transcription activators BMAL1 and CLOCK bind as heterodimers to E-box promoter elements present in *Period* (*Per*) and *Cryptochrome* (*Cry*) genes and activate their transcription. In turn, PER and CRY proteins accumulate in the nucleus and inhibit BMAL1/CLOCK mediated transactivation. An additional essential feedback loop involves the orphan nuclear receptors of the REV-ERB and ROR families. BMAL1 activates *Rev-Erb* transcription, which in turn suppresses *Bmal1, Per, Cry* and *Rev-Erb* expression (6–9).

The pervasive involvement of well-characterized large structural domains in various protein interactions within the clock circuitry has been highlighted by plentiful structural and biochemical studies. For example, the structure of the BMAL1/CLOCK heterodimer consists of a basic Helix Loop helix domain that facilitates BMAL1/CLOCK DNA-binding and PER-ARNT-SIM domain, necessary for PER binding as well as interactions with additional regulatory proteins (10,11). Similarly, CRY and PER harbor distinct protein domains that are critical for their dimerization, the transcriptional repression of BMAL1/CLOCK and binding of their ubiquitin ligases (12–17).

Conventionally, large structural protein domains were considered as major mediators of protein interactions. However, accumulating evidence has revealed that disordered protein regions, in particular short linear motifs, also participate in modulation of protein interactions (18). Short linear motifs consist of 3–11 amino acids that exhibit evolutionary plasticity and participate in low affinity, transient and highly dynamic protein interactions (19). They play critical roles in protein-complex formation, posttranslational modifications and transcription regulation (20,21). Hitherto, the involvement of protein linear motifs in the clock's function was largely overlooked. The best-documented example is the LXXLL motif of PER2 that participates in PER2 interactions with various nuclear receptors and plays a role in circadian gene expression (22).

In this study, we performed a bioinformatics analysis of short linear motifs, implicated in protein interactions and identified an enrichment of the Pro-X-Asp-Leu-Ser (PXDLS) motif within circadian transcripts. We found that the PXDLS motif can bind to BMAL1/CLOCK and disrupt circadian oscillations in cultured cells in a cell-autonomous manner. Remarkably, the PXDLS motif is evolutionary conserved in the core clock protein REV-ERBα, and additional proteins implicated in the clock's function (Nuclear Receptor Interacting Protein 1 (NRIP1) and Creb-Binding Protein (CBP)). In this conjuncture, we discovered that BMAL/CLOCK and REV-ERBα physically interact in a PXDLS-dependent manner. Furthermore, we revealed that the PXDLS motifs of NRIP1 and CBP are involved in circadian rhythmicity. Taken together, our results suggest that the PXDLS motif plays a role in circadian rhythmicity through regulation of protein interactions within the core clock circuitry.

## MATERIALS AND METHODS

### Bioinformatics analysis

Fuzzpro in the EMBOSS package (23) was used for pattern searches. The mouse whole proteome dataset was taken from UniProt (22,597 non-redundant gene symbols). Enrichment calculations were made in respect to liver 24 h circadian expression dataset (24) (2777 circadian out of a total possible 21,865 non-redundant gene symbols). The enrichments were calculated using ratio comparison as follows: for the enrichment of the motif within circadian expression dataset, the number of genes with a given motif was divided into the total number of genes (general population ratio), and compared to the number of circadian genes with the same motif divided by the total number of circadian genes (circadian ratio). The enrichment is [(circadian ratio–general population ratio)/general population ratio] * 100, to reach a percent enrichment. Likewise, the enrichment of circadian genes within genes containing the motif was calculated as follows: the ratio of circadian genes to all genes (general circadian ratio) was compared to the ratio of motif containing circadian genes divided by all motif containing genes (motif ratio). The enrichment is [(motif ratio–general circadian ratio)/general circadian ratio] * 100.

Sequences used for alignment: REV-ERBα (R1D1): Human-NP_068370, Mouse-NP_663409, Chinchilla lanigera-XP_005394402, Drosophila melanogaster-NP_001246822; CPB (CREBBP): Human-NP_004371, Mouse-NP_001020603, Chicken XP_414964, Zebrafish-XP_003198104; NRIP1: Human-NP_003480, Mouse-NP_775616, Chicken-XP_004938408, Zebrafish- XP_005157805.

### Mice

All animal experiments and procedures were conducted in conformity with the Institutional Animal Care and Use Committee guidelines. Liver nuclear extracts were prepared from C57BL/6 mice as previously described (25).

### Cell culture

HEK 293T, NIH3T3 and NIH3T3-Rev-VNP (26) cells were grown in Dulbecco's modified Eagle's medium supplemented with 10% FBS, 100 units/ml penicillin, 100 mg/ml streptomycin and cultured at 37°C in a humidified incubator with 5% CO_2_. Transfections of HEK 293T cells were performed with BES-CaCl_2_ and of NIH3T3 cells with jetPEI (Polyplus Transfection).

### Plasmids and cloning

The following plasmids were used: *Bmal1*-luciferase, *Dbp*-luciferase, CMV-luciferase (27), pLA-CMV mBMAL1-Flag, pCM mREV-ERBα-Myc (kindly provided by H. Reinke), pCIEF-hNRIP1-V5 (kindly provided by M. Parker), pcDNA-PER2-Flag, pCAGGS-mCherry, pRc/RSV-mCBP-HA (Addgene, 16701). pIRES Flag-PXDLS*3 and pIRES Flag-Mut PXDLS*3 (i.e. ASASA*3) were cloned from pMI G-ZEB700-776 and pMI G-ZEB mut 700–776 respectively (28). The different linear motifs, namely PXDLS*3, Mut PXDLS*3 synthetic PXDLS, synthetic Mut PXDLS, RGD*3, PPXY*3, TRAF6*3, NPF*3 and PTAP*3, were cloned into pIRES expression vector and their peptide sequences are detailed in Supplementary Table S2. The hNRIP1 P440A, mCBP P1133A and mREV-ERBα P72A mutants were generated by site directed mutagenesis using Restriction-free cloning.

### Recombinant peptides and protein preparation

PXDLS*3 and mutant PXDLS*3 were cloned into pGEX-4T-1. The plasmids pGEX-4T-1, pGEX-4T-1 PXDLS and pGEX-4T-1 mutant PXDLS*3, were expressed in *Escherichia coli* BL21 (DE3) pLysS using the IPTG induction system. Similarly, pET15B-6xHis and pET15B-6xHis-mBMAL1 (kindly provided by J. A. Ripperger) were expressed in *E. coli*. GST-fused peptides and His-tagged proteins were purified by affinity resin, using glutathione agarose and HIS-Select Nickel Affinity beads (Sigma) respectively. GST-PXDLS*3 and GST-Mut PXDLS*3 were released from the resin with 10 mM reduced glutathione (Sigma) in 50 mM Tris HCl, pH 9.5.

### Pull down assay with *in vitro* translated labeled proteins

[35S] methionine labeled REV-ERBα was *in vitro* translated using TnT Quick Coupled Transcription/Translation System (Promega) according to the manufacturer's instructions. Pull down assays were performed with recombinant proteins coupled to beads, incubated with *in vitro* [35S] methionine labeled REV-ERBα, for 3 h at room temperature and washed with NP40 buffer (150 mM NaCl, 100 mM Tris pH-7.5, 2 mM EDTA, 1%NP40). Samples were analyzed by sodium dodecyl sulphate-polyacrylamide gel electrophoresis (SDS-PAGE), recombinant proteins were detected by Coomassie stain and [35S] labeled REV-ERBα by autoradiography.

### Real-time bioluminescence monitoring

Cells were seeded on 35 mm culture dishes at 70% confluence and transfected with the indicated reporter plasmids. Cells were synchronized with 100 nM dexamethasone treatment for 20 min and bioluminescence was recorded using the LumiCycle (Actimetrics). Data were analyzed with the LumiCycle analysis software.

### Single cell analysis

NIH3T3-Rev-VNP cells were plated on a 96-well plate and transfected with Cherry expression vector either with the PXDSL*3 or the mutant PXDSL*3 transgene. Cells were synchronized with 100 nM dexamethasone and fluorescence was monitored using time-lapse fluorescence microscopy for 72 h at 1 h resolution. Single cells expressing Cherry and neighboring cells were followed and analyzed for VNP fluorescence intensity using the Circadian Gene Express software (29). To reduce cell toxicity, the mCherry signal for transfection validation was acquired only at the beginning and the end of the experiment, while the yellow fluorescent protein (YFP) signal was followed throughout the entire experiment.

### Protein extraction and immunoblotting

Cells were collected and homogenized in RIPA buffer as previously described (27). SDS-PAGE and immunoblot were performed according to standard procedures. Antibodies used: rabbit anti CRY1, PER2, BMAL1, CLOCK, Rap1 (27), REV-ERBα (Cell signaling) and GST (Sigma). Mouse anti V5 (Invitrogen), Flag and U2AF (Sigma). PageRuler prestained protein ladder 10–170 kd (Pierce) was used as a protein size marker.

### Co-immunoprecipitation experiments

Experiments were carried out from mouse liver nuclear extracts or from cultured cell extracts as specified. Protein extracts were incubated with indicated antibodies for 12 h at 4°C, and subsequently incubated with pre-blocked protein A/G agarose beads (Santa Cruz) for an additional 1 h at 4°C. For immunoprecipitation of Flag tagged proteins, extracts were incubated with pre-blocked Flag antibody conjugated beads (Sigma). Beads were washed with RIPA buffer, and samples were mixed with Laemmli sample buffer and heated at 95°C for 5 min. For the purification of the Flag tagged peptide or in case more stringent conditions were required the beads were washed three times with RIPA buffer, subsequently four washes with B Buffer (100 mM KCl, 0.5 mM EDTA, 20 mM Hepes pH-7.6, 0.4%NP40, 20% Glycerol) and finally twice with 54K Buffer (150 mM NaCl, 50 mM Tris pH-7.9, 0.5%Tryton). The Flag tagged peptides were eluted with 100 μg/ml of Flag peptide (Sigma) in Tris Buffeed Saline.

## RESULTS

### PXDLS-containing proteins are enriched within circadian transcripts

**Figure 1.**
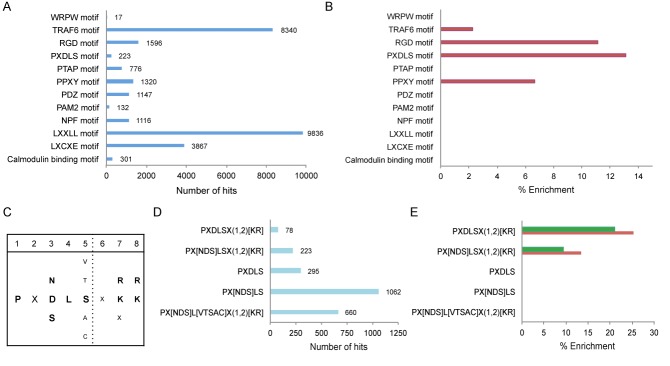
PXDLS-containing proteins are enriched within circadian transcripts. (**A**) Pattern search for the indicated short linear motifs in mouse whole proteome dataset (UniProt). (**B**) Protein populations containing the indicated short linear motif were analyzed for enrichment within circadian expression dataset (24). (**C**) Consensus sequences of the PXDLS motif with its various variants. (**D**) Pattern search for the indicated variants of the PXDLS motif in mouse whole proteome dataset. (**E**) Protein populations containing the indicated variants of the PXDLS motif were analyzed for enrichment within circadian expression dataset (red). Circadian expression dataset was tested for enrichment within population of proteins containing the indicated variants of the PXDLS motif (green). The enrichments were calculated using ratio comparison as described in Materials and Methods.

To obtain an insight into the potential involvement of protein linear motifs in circadian rhythms, we have screened a dozen well-characterized short motifs implicated in protein–protein interactions (Eukaryotic Linear Motifs database) (30) and examined their propensity within a circadian expression dataset (24). The following linear motifs were analyzed: the WRPW motif ([WFY]RP[WFY]) that facilitates the recruitment of the co-repressors Groucho/transducin-like enhancer-of-split family; the TRAF6 motif (PX[QE]XX[DFYWE]) that mediates interactions with different cellular receptors; the RGD motif (RGD), which is implicated in ligand recognition of extracellular proteins; the PXDLS motif (PX[NDS]LSX(1,2)[KR]), which participates in the recruitment of various transcription regulatory proteins; the PTAP motif (XP[TS]APX) that modulates various cellular protein–protein interactions; the PPXY motif (PPXY) that is involved in binding to WW domains; the PDZ-binding motif (X[ST]X[LV]) that is implicated in recognition of the PDZ domain; the PAM2 motif (SX[LFP]NXXAXXF), which participates in interactions of polyadenylate binding proteins; the NPF motif (XNPFX) that binds to EH domains; the LXXLL motif ([∧P]L[∧P][∧P]LL[∧P]), which facilitates the binding of transcription regulators to nuclear receptors; the LXCXE motif ([LI]XCX[DE]) that mediates recruitments of different regulatory proteins and the Calmodulin binding motif ([ILV]QXXXRXXXX[RK]XX[FILVWY]), participating in the interaction of calcium-binding proteins. Their prevalence based on pattern search in the whole mouse proteome was 17, 8340, 1596, 223, 776, 1320, 1147, 132, 1116, 9836, 3867 and 301 non-redundant genes respectively (Figure 1A).Both the TRAF6 and PPXY motifs were slightly enriched (2.2% and 6.6% respectively), whereas the RGD and the PXDLS motifs exhibited >10% enrichment among circadian transcripts (Figure 1B).

The enrichment of the PXDLS motif within circadian transcripts incited us to look deeper at this specific motif. Though, historically it was termed as the PXDLS motif, during the last decades various functional assays evinced that the motif encompasses a wider definition that is not necessarily represented by its original name (28,31). In its broad definition the PXDLS motif contains several variants (Figure 1C) including the PX[NDS]LSX(1,2)[KR] pattern that we employed for the initial bioinformatics screen (30). Thus, we use the common nomenclature and refer to the motif in its broad definition as the PXDLS motif and the different options as variants. Next, we performed a bioinformatics analysis specifically addressing the different variants of the PXDLS motif. These include PX[NDS]L[VTSAC]X(1,2)[KR], PX[NDS]LS, PXDLS, PX[NDS]LSX(1,2)[KR] and PXDLSX(1,2)[KR]. Their prevalence in the whole mouse proteome was 660, 1062, 295, 233 and 78 non-redundant genes respectively (Figure 1D). We identified a significant enrichment of proteins containing the stringent patterns PX[NDS]LSX(1,2)[K,R], and PXDLSX(1,2)[KR], (13% and 25% respectively) among circadian transcripts (Figure 1E). By contrast, the PXDLS and PX[NDS]LS variants lacking the lysine/arginine residues in the last two positions were not enriched, suggesting that these specific residues might have a critical role. We also performed the reciprocal analysis and found that circadian transcripts are enriched among PX[NDS]LSX(1,2)[KR], and PXDLSX(1,2)[KR] containing proteins (9.5% and 21% respectively) (Figure 1E). For a detailed list of circadian transcripts that contain the different variants of PXDLS motif see Supplementary Table S1.

Taken together, our bioinformatics analysis evinced that the PXDLS motif is more prevalent among transcripts that are expressed in a circadian manner and that transcripts containing this motif have higher likelihood to oscillate; hence underscoring a possible link between the PXDLS motif and circadian rhythmicity.

### The PXDLS motif affects circadian rhythmicity

Our findings that transcripts harboring certain linear motifs, in particular the PXDLS motif, are more likely to be expressed in a circadian manner prompted us to explore the prospective role of these motifs in circadian rhythmicity. To this aim we monitored circadian oscillations in living cells applying a cell-culture-based assay with a short-lived luciferase reporter that is driven by a circadian promoter (i.e. *Bmal1*-luciferase) (26). Circadian clocks in different individual cultured cells were synchronized with a short pulse of dexamethasone treatment and bioluminescence was recorded using a photomultiplier tube, as previously described (26). We employed several different transgenes encoding three repeats in tandem of each linear motif separated by short spacers. Co-expression of the different linear motif transgenes together with the *Bmal1*-luciferase reporter evinced that TRAF6, PPXY, RGD and PXDLS reduced the amplitude of the circadian oscillations, while NPF and PTAP motifs had no significant effect on circadian rhythmicity (Supplementary Figure S1). These findings correlated well with our bioinformatics analysis as the former motifs (i.e. TRAF6, PPXY, RGD and PXDLS) were enriched among circadian transcripts and altered circadian rhythmicity, whereas the latter motifs (i.e. NPF and PTAP) were not enriched and did affect the circadian oscillations.

**Figure 2.**
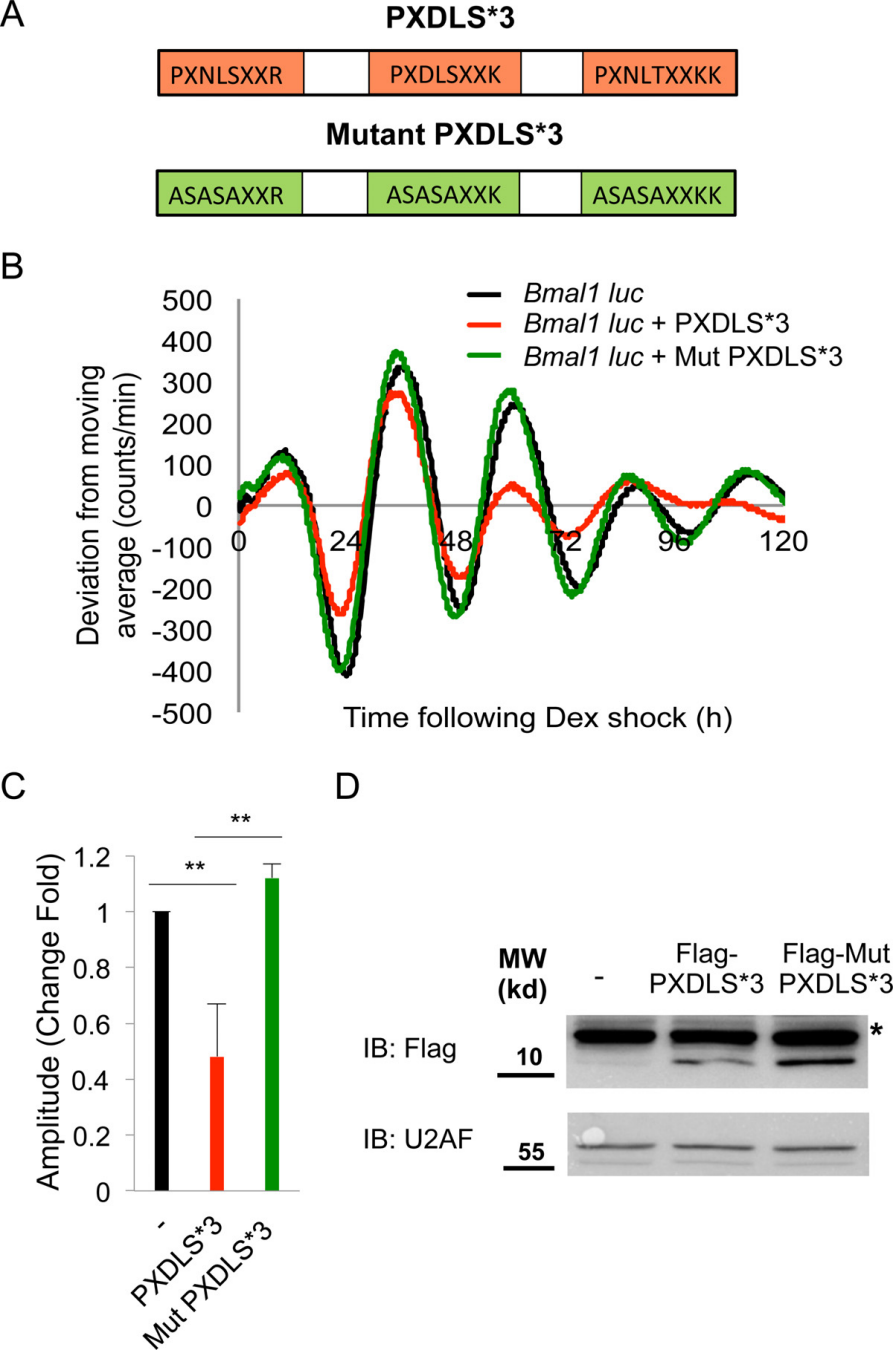
The PXDLS motif affects circadian rhythmicity. (**A**) Schematic depiction of the PXDLS*3 (red) and the mutant PXDLS*3 (green) peptides. (**B**) NIH3T3 cells were transiently transfected with *Bmal1*-luciferase reporter either alone (black) or together with the PXDLS*3 (red) or the mutant PXDLS*3 (green) transgene. Cells were synchronized with a short dexamethasone (Dex) treatment, and real-time bioluminescence recording were performed using the LumiCycle. (**C**) Amplitudes were quantified using the LumiCycle software. Data are presented on a bar graph as fold change, (mean +/− SD, *n* = 4), *P*-values <0.001 is marked with **. (**D**) Expression levels of the PXDLS*3 and the mutant PXDLS*3 peptides were determined by SDS-PAGE and immunoblot (IB). *Non-specific band, Molecular Weight (MW).

Our results that transcripts containing the PXDLS motif are more likely to be circadianly expressed and vice versa, together with the prominent effect of the PXDLS motif on circadian oscillations encouraged us to further explore in detail the prospective role of this specific motif in circadian rhythmicity. The aforementioned PXDLS peptide contained three different variants of the PXDLS motif in tandem, namely PLNLSXXR, PLDLSXXK and PLNLTXXKK, separated by short spacers (PXDLS*3 transgene). To confirm that the effect is dependent on the PXDLS motif, we used a mutant peptide, in which all three variants of the PXDLS motif were mutated to ASASA (mutant PXDLS*3 transgene) (Figure 2A). Co-expression of the PXDLS*3 but not the mutant PXDLS*3 transgene together with the *Bmal1*-luciferase reporter resulted in a significant reduction in the amplitude of the circadian oscillations, indicating that the effect is PXDLS-dependent (Figure 2B and C). The expression levels of both the PXDLS*3 and the mutant PXDLS*3 peptides were similar (Figure 2D). Similar results were obtained with the *Dbp*-luciferase circadian reporter (Supplementary Figure S1A and B). A comparable effect was achieved with a different peptide that contains PXDLS motifs in tandem separated by Flag epitopes (synthetic PXDLS) (Supplementary Figure S2C, D and E), excluding the significance of specific spacers in the effect of the PXDLS motif. The above-described experiments suggested that an intact PXDLS linear motif in tandem is both required and sufficient to disrupt circadian rhythmicity in a cell population.

### The PXDLS motif disrupts circadian rhythmicity in a cell-autonomous manner

**Figure 3.**
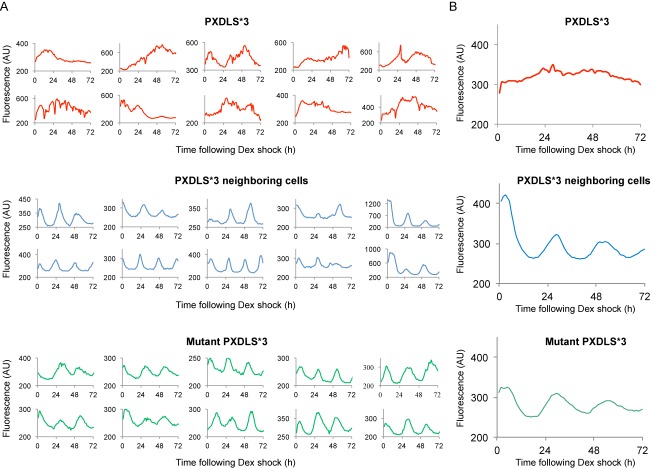
The PXDLS motif disrupts circadian rhythmicity in single cells. (**A**) NIH3T3 cells stably expressing Venus-REV-ERBα reporter were transfected with Cherry expression vector either with the PXDLS*3 or the mutant PXDLS*3 transgene. Cells were synchronized with a short Dex treatment and single cells either expressing the Cherry fluorescent protein (i.e. PXDLS*3 or mutant PXDLS*3) or non-expressing cells (PXDLS*3 neighboring cells) were monitored and analyzed for their circadian oscillations using time-lapse fluorescence microscopy for three consecutive days at 1 h resolution. Each plot represents fluorescence intensity profile for an individual cell (for additional plots see also Supplementary Figure S3). (**B**) An average plot of single cell profiles expressing the PXDLS*3 (red), the mutant PXDLS*3 transgene (green) or non-expressing cells (PXDLS*3 neighboring cells) (blue) (*n* = 25 for each condition). Arbitrary Units (AU).

Hitherto, the involvement of the PXDLS motif was assessed in a cell population, causing shallow circadian oscillations. Low-amplitude oscillations in a cell population might be due to inefficient synchronization of circadian clocks within individual cells that leads to rapid dampening, or the result of an inherent cell-autonomous defect in the clock's function. To discriminate between the two scenarios, we performed fluorescence recording of NIH3T3-Rev-VNP cells, stably expressing the circadian Venus-Rev reporter (26). Cells were transfected with Cherry expression vector either with the PXDLS*3 or the mutant PXDLS*3 transgene as a control. Single cells expressing the Cherry fluorescent protein or neighboring cells that do not express the Cherry fluorescent protein were monitored and analyzed for their circadian oscillations (Figure 3, Supplementary Figure S3, and Supplementary Movie S1). In contrast to the mutant PXDLS*3 and the PXDLS*3 non-expressing cells (neighboring cells), overexpression of the PXDLS*3 transgene disrupted the circadian rhythmicity in single cells (Figure 3, Supplementary Figure S3, and Supplementary Movie S1).

The above-described experiments demonstrated the prominent effect of a short PXDLS peptide on circadian oscillations and suggested that the PXDLS motif might play a regulatory role in circadian rhythmicity in a cell-autonomous manner. It is conceivable that the striking phenotype we observed in single cells, compared to a cell population, was because in single cells we monitor highly PXDLS expressing cells based on the Cherry fluorescent signal, whereas in a cell population analysis the transgene expression levels vary between the different cells.

### The core clock proteins BMAL1 and CLOCK bind to the PXDLS motif.

**Figure 4.**
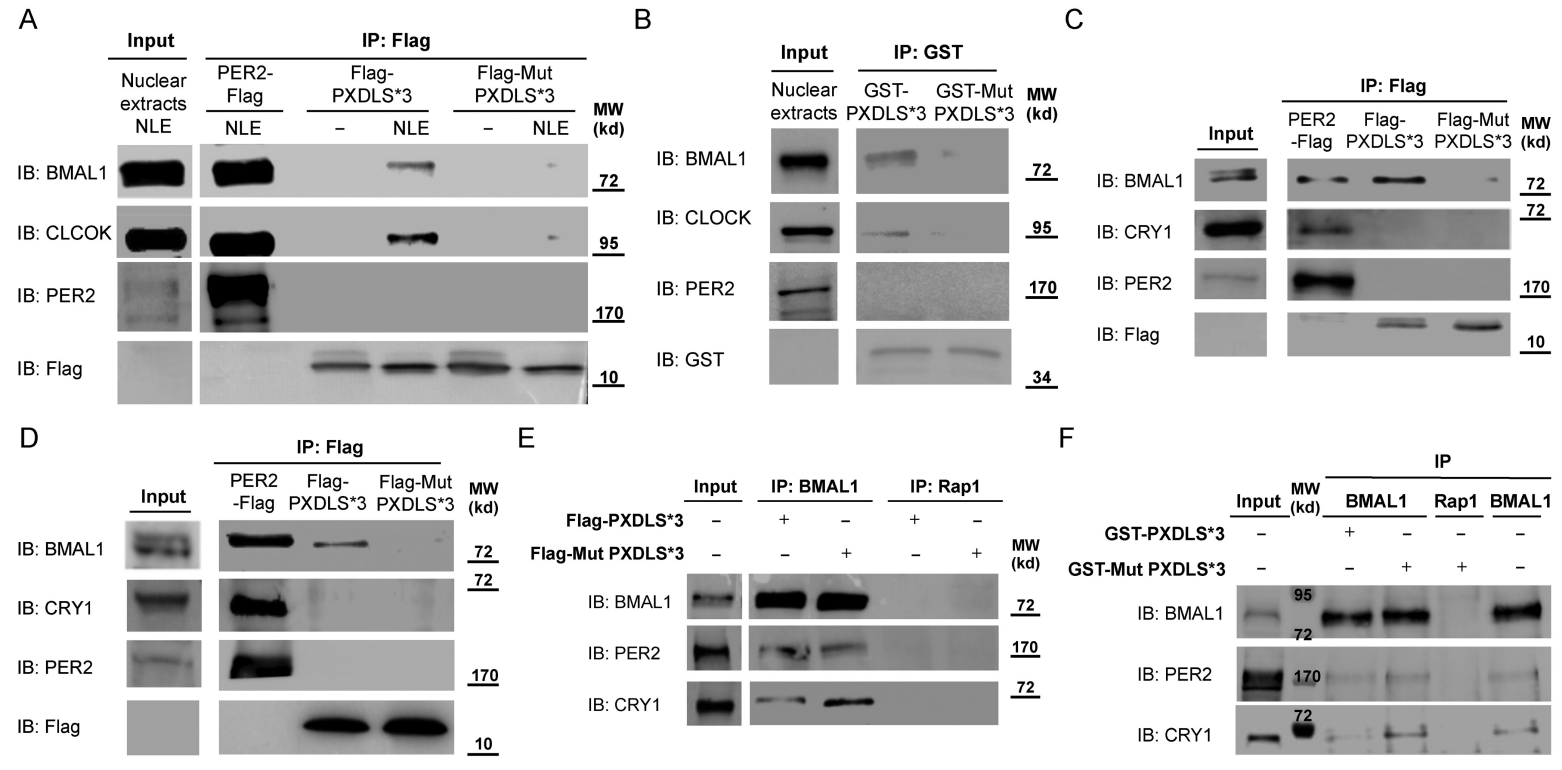
BMAL1 and CLOCK bind to the PXDLS motif. (**A**) PER2-Flag, Flag-PXDLS*3 or Flag-Mut PXDLS*3 transgenes were expressed in HEK 293T cells; protein lysates were prepared and immunoprecipitated (IP) with Flag antibody conjugated beads. The beads were extensively washed with stringent washes (see Materials and Methods) and subsequently incubated without or with mouse liver nuclear extracts (NLE). The pulled down proteins were analyzed by SDS-PAGE and IB. (**B**) Recombinant GST-PXDLS*3 and GST-Mut PXDLS*3 coupled to glutathione beads were incubated with mouse liver nuclear extracts (input). The pulled down proteins were analyzed by SDS-PAGE and IB. (**C**) PER2-Flag, Flag-PXDLS*3 or Flag-Mut PXDLS*3 transgenes were expressed in NIH3T3 cells; protein lysates were prepared and immunoprecipitated with Flag antibody conjugated beads. Endogenous co-immunoprecipitated proteins were analyzed by SDS-PAGE and IB. (**D**) PER2-Flag, Flag-PXDLS*3 or Flag-Mut PXDLS*3 transgenes were expressed in HEK 293T cells; protein lysates were prepared and immunoprecipitated with Flag antibody conjugated beads. Endogenous co-immunoprecipitated proteins were analyzed by SDS-PAGE and IB. (**E**) BMAL1 was immunoprecipitated from mouse liver nuclear extracts using BMAL1-specific antibody, in the presence of either purified Flag-PXDLS*3 or Flag-Mut PXDLS*3 peptide. Immunoprecipitated proteins were analyzed by SDS-PAGE and IB. (**F**) BMAL1 was immunoprecipitated from mouse liver nuclear extracts using BMAL1-specific antibody, in the presence of either recombinant GST-PXDLS*3 or GST-Mut PXDLS*3 peptide. Immunoprecipitated proteins were analyzed by SDS-PAGE and IB. Rap1 antibody was used as negative control. Molecular Weight (MW).

The PXDLS motif plays a major role in modulating protein interactions (31,32). Since the core clock circuitry relies on multiple protein interactions, we tested the possible involvement of the PXDLS motif in these interactions. We performed pull down experiments using purified Flag tagged PXDLS*3 or mutant PXDLS*3 peptide as a bait (Figure 4A). PER2-Flag was used as a positive control, known to bind different core clock components. Flag tagged PXDLS*3, mutant PXDLS*3 and PER2 were expressed and purified from HEK 293T cells, using Flag antibody conjugated beads. The beads were extensively washed with relatively stringent washes to reduce the co-purification of interacting proteins from the HEK 293T cells and subsequently incubated with mouse liver nuclear extracts. Both BMAL1 and CLOCK were specifically pulled down with Flag-PXDLS*3 and PER2-Flag, but not with Flag-mutant PXDLS*3 peptide (Figure 4A). Similar results were obtained with bacterially expressed and purified glutathione coupled GST-PXDLS*3 and GST-mutant PXDLS*3 peptides (Supplementary Figure S4). Both BMAL1 and CLOCK were specifically pulled down from mouse liver nuclear extract with GST-PXDLS*3, but not with GST-mutant PXDLS*3 recombinant peptide (Figure 4B).

To examine the *in vivo* interactions between the PXDLS motif and core clock proteins, we performed immunoprecipitation experiments in cultured cells (NIH3T3 and HEK 293T) expressing Flag-PXDLS*3, Flag-mutant PXDLS*3 and PER2-Flag constructs (Supplementary Figure S5A and B). In NIH3T3 cells, endogenous BMAL1 co-immunoprecipitated with the PXDLS*3 but not with the mutant PXDLS*3 peptide (Figure 4C). Similar results were obtained in HEK 293T cells, as endogenous BMAL1 co-immunoprecipitated with the PXDLS*3 but not with the mutant PXDLS*3 peptide (Figure 4D). Neither CRY1 nor PER2 co-immunoprecipitated with the PXDLS*3 peptide in both cell lines (Figure 4C and D).

Given the possibility that BMAL1/CLOCK heterodimers bind the PXDLS motif, we tested whether the PXDLS motif might interfere with their binding to known partners (e.g. PER2 and CRY1). First, we purified Flag tagged PXDLS*3 and mutant PXDLS*3 peptides from HEK 293T cells (Supplementary Figure S6). BMAL1 was immunoprecipitated from liver nuclear extracts, in the presence of purified Flag tagged PXDLS*3 or mutant PXDLS*3 peptide. As expected, both CRY1 and PER2 co-immunoprecipitated with BMAL1. However, in the presence of the PXDLS*3 peptide, we observed a decrease in the binding of CRY1 whereas PER2 binding was unaffected (Figure 4E). Comparable results were obtained with recombinant GST-PXDLS*3 and GST-mutant PXDLS*3 peptides (Supplementary Figure S4), as in the presence of GST-PXDLS*3 there was a decrease in the binding of CRY1 to BMAL1 (Figure 4F and Supplementary Figure S7).

These experiments pointed out that BMAL1 and CLOCK can bind the PXDLS motif, both *in vitro* and *in vivo*, and that the PXDLS peptide can modulate protein–protein interactions within core clock complexes, such as the interaction between BMAL1/CLOCK and CRY1, and thus disrupt circadian rhythmicity.

### BMAL1/CLOCK interact with REV-ERBα

**Figure 5.**
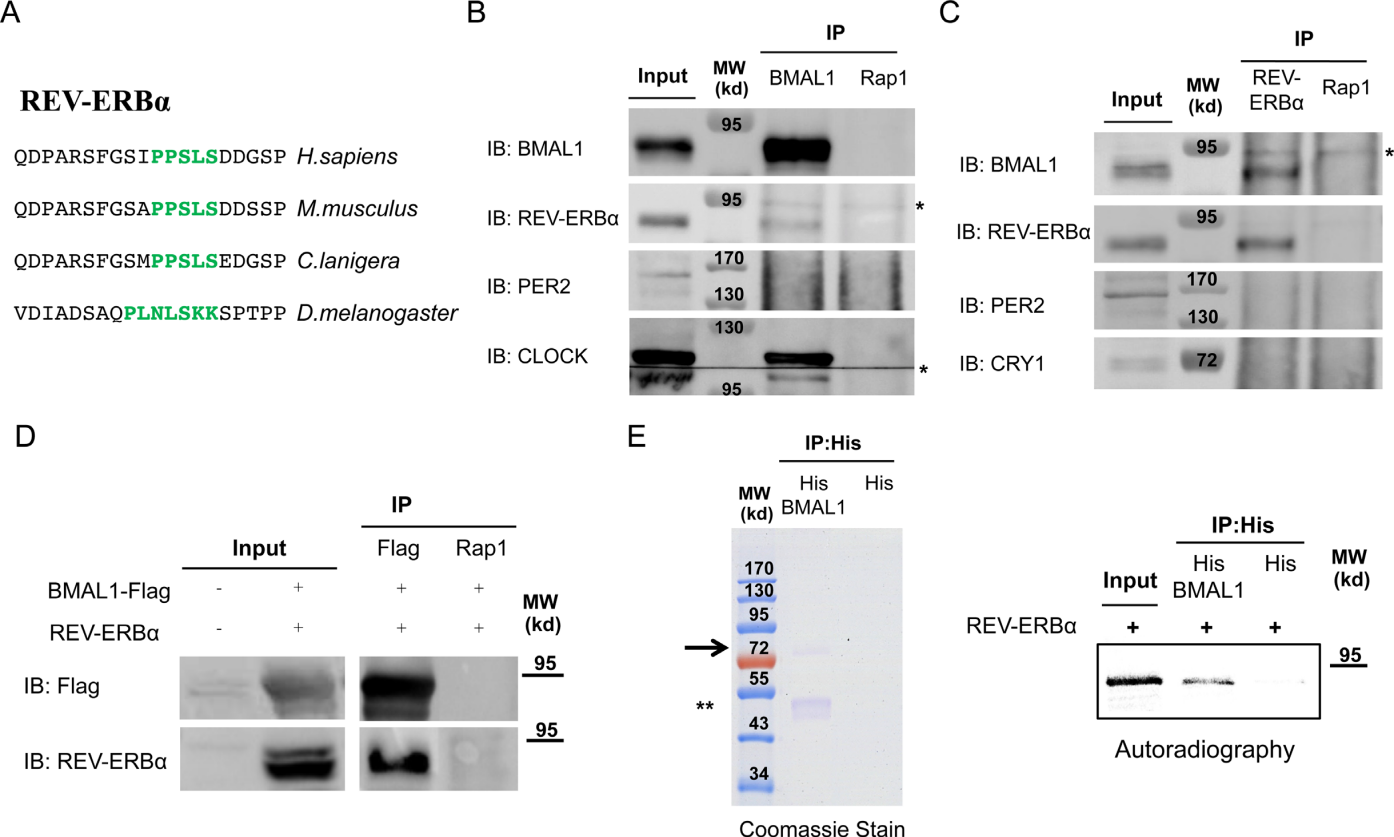
BMAL1/CLOCK interact with REV-ERBα. (**A**) Schematic representation of the evolutionary conservation of the PXDLS motif (green) in REV-ERBα. Mice were sacrificed at Zeitgerber 4 (ZT4), 4 h after lights were turned on in the animal facility, livers were harvested and nuclear extracts were prepared. Immunoprecipitation (IP) experiments were performed with anti BMAL1 (**B**) or anti REV-ERBα (**C**) antibodies. Rap1 antibody was used as negative control. Immunoprecipitated proteins were analyzed by SDS-PAGE and IB. (**D**) BMAL1-Flag and REV-ERBα were expressed in NIH3T3 cells; protein lysates were prepared and immunoprecipitated with Flag or Rap1 antibody. Immunoprecipitated proteins were analyzed by SDS-PAGE and IB. (**E**) Pull down assay with recombinant His-BMAL1 coupled to beads and *in vitro* translated [35S] labeled REV-ERBα. Recombinant His-BMAL1 was detected by Coomassie stained SDS-PAGE and [35S] labeled REV-ERBα by autoradiography. Molecular Weight (MW). *Non-specific band. An arrow indicates the full length BMAL1. **A degradation product of recombinant BMAL1.

To uncover the molecular mechanisms underlying the involvement of the PXDLS motif in circadian oscillations, we searched for proteins containing the PXDLS motif among proteins that play a role in circadian rhythmicity. Notably, we found that the motif is evolutionary conserved in the core clock protein, REV-ERBα (Figure 5A), that participates in the repression of *Bmal1* promoter (6–9). The conserved PXDLS domain in REV-ERBα on the one hand, and our findings that BMAL1/CLOCK can bind the PXDLS motif on the other hand, prompted us to examine whether REV-ERBα interacts with BMAL1/CLOCK. Thus, we performed co-immunoprecipitation experiments from liver nuclear extracts. Immunoprecipitation of BMAL1 resulted in co-immunoprecipitation of endogenous REV-ERBα and CLOCK (Figure 5B, Supplementary Figures S8A and S9A). Likewise, both endogenous BMAL1 and CLOCK co-immunoprecipitated with REV-ERBα (Figure 5C, Supplementary Figures S8B and S9B). Similar interaction was detected in NIH3T3 cells, upon co-expression of BMAL1-Flag and REV-ERBα and immunoprecipitation of BMAL1-Flag (Figure 5D). Finally, to test whether this interaction can be recapitulated *in vitro*, we prepared recombinant BMAL1 and examined its binding to *in vitro* translated [35S] labeled REV-ERBα. REV-ERBα was specifically pulled down in the presence of recombinant BMAL1 (Figure 5E).

Our data showed that BMAL1/CLOCK and REV-ERBα interact both *in vivo* (i.e. in mouse liver nuclear extracts and in cultured cells) and *in vitro* (i.e. recombinant and *in vitro* translated labeled protein).

### The PXDLS motif plays a role in BMAL1/CLOCK–REV-ERBα interaction

**Figure 6.**
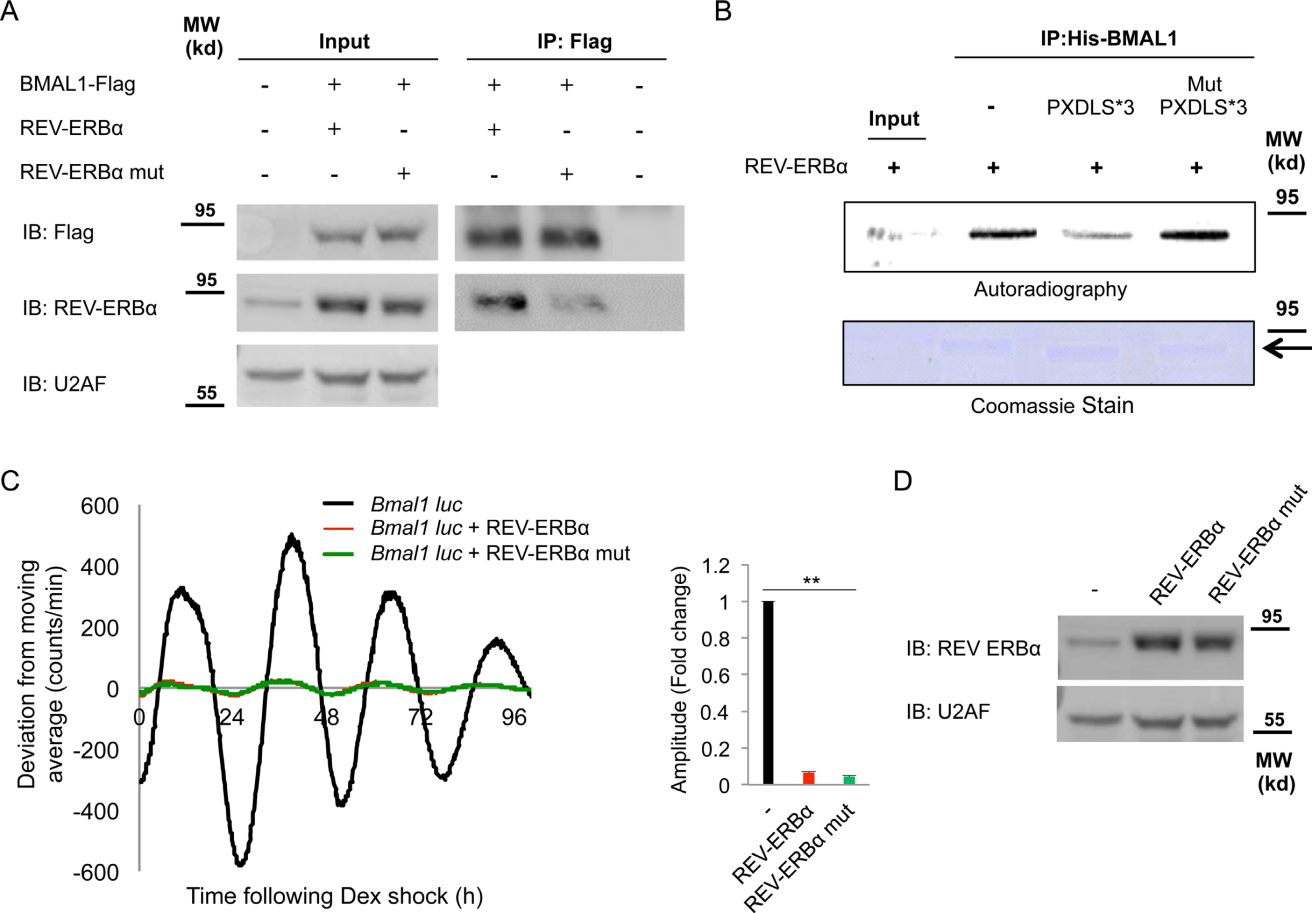
The PXDLS motif plays a role in BMAL1/CLOCK–REV-ERBα interaction. (**A**) NIH3T3 cells were transfected with BMAL1-Flag either with wild-type REV-ERBα or with PXDLS mutant REV-ERBα (REV-ERBα P72A) or non-transfected; protein lysates were prepared and immunoprecipitated with Flag antibody. Immunoprecipitated proteins were analyzed by SDS-PAGE and IB. (**B**) Pull down assay with recombinant His-BMAL1 coupled to beads and *in vitro* translated [35S] labeled REV-ERBα in the presence or absence of recombinant GST-PXDLS*3 or GST-Mut PXDLS*3 peptide. Recombinant BMAL1 was detected by Coomassie stained SDS-PAGE and [35S] labeled REV-ERBα by autoradiography. (**C**) NIH3T3 cells were transiently transfected with *Bmal1*-luciferase reporter either with control empty vector (black) or with wild-type REV-ERBα (red) or PXDLS mutant REV-ERBα (green). Cells were synchronized with a short dexamethasone (Dex) treatment, and real-time bioluminescence recording was performed using the LumiCycle. Amplitudes were quantified using the LumiCycle software. Data are presented on a bar graph as fold change (mean +/− SD, *n* = 4), *P*-values <0.001 is marked with **. (**D**) The expression levels of wild-type REV-ERBα and PXDLS mutant REV-ERBα were determined by SDS-PAGE and IB. U2AF was used as loading control. Molecular Weight (MW). An arrow indicates the full length BMAL1.

To examine the role of the PXDLS motif in the BMAL1/CLOCK–REV-ERBα interactions, we mutated the PXDLS motif of REV-ERBα (REV-ERBα P72A) and examined its binding to BMAL1. Co-immunoprecpitation experiments showed that mutation in the PXDLS motif of REV-ERBα decreased the binding of REV-ERBα to BMAL1 (Figure 6A). To further test the role of the PXDLS motif of REV-ERBα in the interaction, we examined the effect of recombinant PXDLS peptide on the binding of *in vitro* translated [35S] labeled REV-ERBα, to recombinant BMAL1. The binding of *in vitro* translated [35S] labeled REV-ERBα to recombinant BMAL1 declined in the presence of excess of PXDLS but not the mutant PXDLS peptide (Figure 6B).

The evolutionary conservation of the PXDLS motif in REV-ERBα, and the role of the PXDLS motif in BMAL1/CLOCK–REV-ERBα interaction, incited us to test its effect on circadian rhythmicity, and more specifically examine the possible relevance of its PXDLS motif for circadian oscillations. To this aim NIH3T3 cells were transfected with *Bmal1*-luciferase reporter, alone or together with REV-ERBα. As anticipated from its known function as a repressor of the *Bmal1* promoter, overexpression of REV-ERBα resulted in shallow circadian oscillations (Figure 6C, and Supplementary Figure S10). To examine whether the effect on the circadian oscillations was dependent on the PXDLS motif, we examined the effect of the PXDLS mutant of REV-ERBα (REV-ERBα P72A) on *Bmal1*-luciferase reporter. However, we did not observe any significant difference between the effect of wild type and the PXDLS mutant form of REV-ERBα, implying that under this experimental setup the PXDLS motif does not play a significant role in the effect of REV-ERBα on *Bmal1*-luciferase oscillations. The expression levels of REV-ERBα, and its respective PXDLS mutants, namely REV-ERBα P72A, were comparable (Figure 6D).

Hence, we demonstrated that BMAL1/CLOCK and REV-ERBα interact and that the PXDLS motif of REV-ERBα plays a role in their binding. However, both wild type and the PXDLS mutants of REV-ERBα similarly repressed the circadian oscillations of the *Bmal1*-luciferase reporter.

### The PXDLS-containing proteins, NRIP1 and CBP, affect circadian rhythmicity in a PXDLS-dependent manner

**Figure 7.**
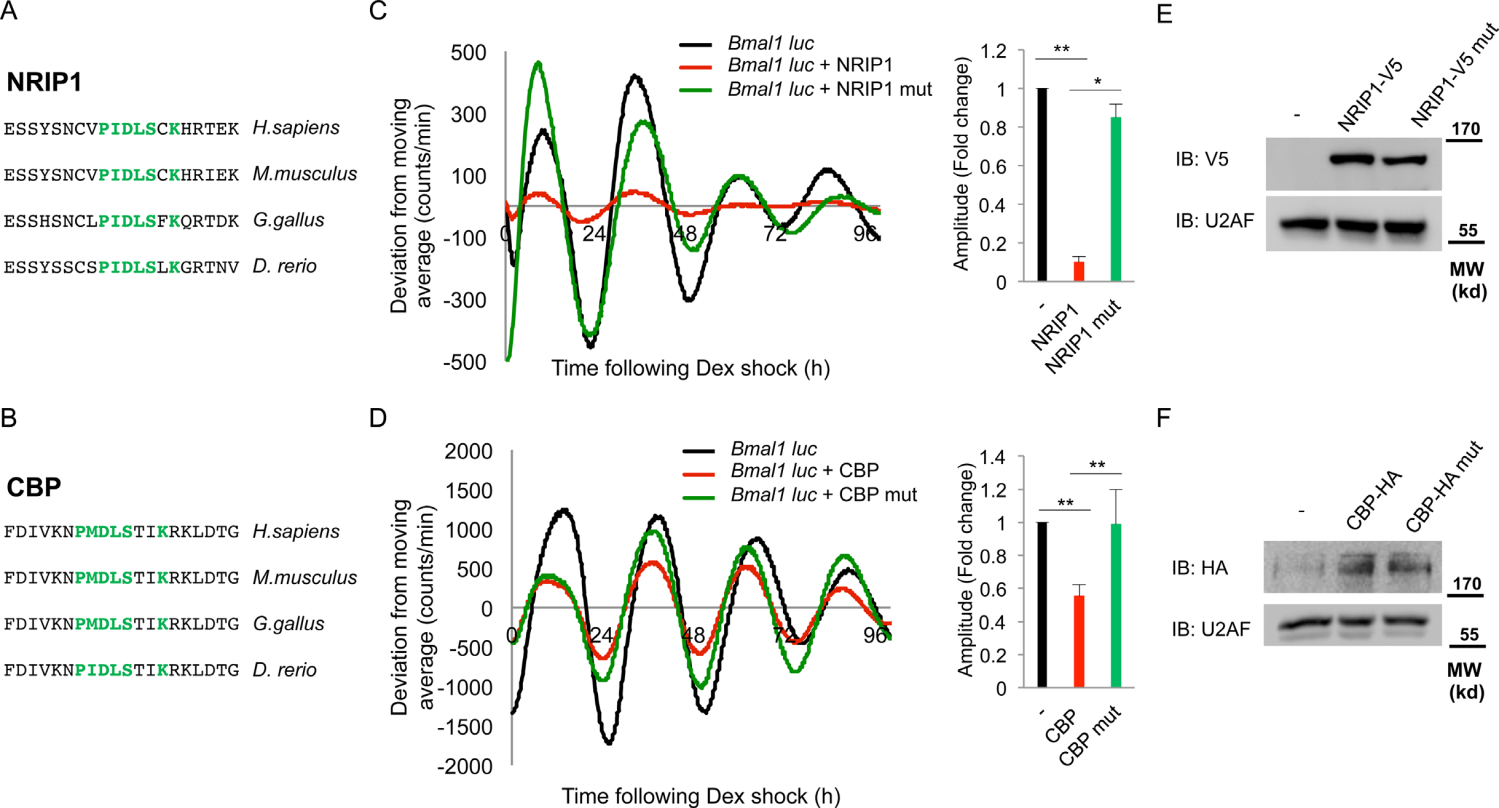
The PXDLS-containing proteins, NRIP1 and CBP, affect circadian rhythmicity in a PXDLS-dependent manner. Schematic representation of the evolutionary conservation of the PXDLS motif (green) in (**A**) NRIP1 and (**B**) CBP. NIH3T3 cells were transiently transfected with *Bmal1*-luciferase reporter either with control empty vector (black) or with (**C**) wild-type NRIP1-V5 (red) or PXDLS mutant NRIP1-V5 (green), (**D**) wild-type CBP-HA (Red) or PXDLS mutant CBP-HA (green). Cells were synchronized with a short dexamethasone (Dex) treatment, and real-time bioluminescence recording was performed using the LumiCycle. Amplitudes were quantified using the LumiCycle software. Data are presented on a bar graph as fold change (mean +/− SD, *n* = 4), *P-*values < 0.001 is marked with ** and <0.005 is marked with *. The expression levels of (**E**) wild-type NRIP1-V5 and PXDLS mutant NRIP1-V5 and (**F**) wild-type CBP-HA and PXDLS mutant CBP-HA were determined by SDS-PAGE and IB. U2AF was used as loading control. Molecular Weight (MW).

In our screen for proteins containing the PXDLS motif among proteins that play a role in circadian rhythmicity, we identified in addition to REV-ERBα, the NRIP1 and CBP (Figure 7A and B). NRIP1 regulates *Bmal1* expression (33), while CBP binds to BMAL1 and is implicated in BMAL1/CLOCK transcriptional co-activation (34). The PXDLS motif is evolutionary conserved in both these proteins (Figure 7A and B).

The presence of the PXDLS motif in NRIP1 and CBP prompted us to test their effect on circadian rhythmicity, and more specifically examine the possible role of their PXDLS motif in circadian oscillations. Overexpression of the PXDLS-containing proteins NRIP1 and CBP resulted in shallow circadian oscillations (Figure 7C and D) (compare black with red curves). This is in agreement with the effect we observed for the PXDLS peptide on the *Bmal1*-luciferase reporter (Figure 2 and Supplementary Figure S1).

To examine whether the effect on the circadian oscillations was dependent on the PXDLS motif, we generated mutants in the PXDLS motifs of NRIP1 (NRIP1 P440A) and CBP (CBP P1133A). We examined the effects of these mutants on circadian oscillations of the *Bmal1*-luciferase reporter and compared them with their wild-type counterparts (Figure 7C, D and Supplementary Figure S10). The PXDLS mutants of NRIP1 and CBP partially restored the circadian oscillations, suggesting that their PXDLS motif plays a role in circadian rhythmicity. Notably, both the wild type and PXDLS mutant versions of NRIP1 and CBP exhibited similar effects on a luciferase reporter driven by a CMV promoter (CMV-luciferase reporter) suggesting that the PXDLS-dependent effect of NRIP1 and CBP might be specific to circadian reporters (Supplementary Figure S11). The expression levels of NRIP1 and CBP and their respective PXDLS mutants, namely NRIP1 P440A and CBP P1133A, were comparable (Figure 7E, F and Supplementary Figure S11).

We concluded that the PXDLS motifs of both NRIP1 and CBP play a role in the function of the core clock oscillator, as their effect on the circadian oscillations in cultured cells was dependent on the presence of an intact PXDLS motif.

## DISCUSSION

Intensive efforts have been dedicated to uncover the molecular architecture of the core clock circuitry. These endeavors have highlighted the basic molecular principle (i.e. transcription-translation feedback loop) that is embedded within the clock's function, yet our knowledge regarding the underlying molecular mechanisms is far from being complete. Hitherto, mounting evidence highlighted the principal role of multiple protein–protein interactions within the core clock circuitry (35) and the pervasive involvement of large structural protein domains in these interactions (1,2). By contrast, the involvement of short linear motifs was largely overlooked.

In this study, we explored the potential role of short linear motifs that are implicated in protein interactions, in circadian rhythms. Specifically, we examined the PXDLS motif, a motif that we first identified based on a bioinformatics analysis to be enriched within circadian transcripts, and that is known to be involved in interactions of transcription factors and transcription regulatory proteins (31,32). It should be noted that our bioinformatics screen of short linear motifs was conducted on the circadian transcriptome. Recent studies comparing transcriptome and proteome data revealed that the levels of many polypeptides encoded by rhythmically expressed mRNAs may not oscillate at significant levels (due to long half-lives), while some proteins produced by constantly expressed transcripts do cycle in abundance (36–38). However, since the circadian proteome landscape is still in its infancy, as both the sensitivity and accuracy of current MS are still lower compared with the standards for mRNA profiling and protein detection is still biased to high-abundance long-lived cytosolic proteins, as reflected by the absence of numerous transcription factors, including core clock and clock regulatory proteins in these studies, we conducted our bioinformatics screen on the circadian transcriptome. Thus, to render our bioinformatics analysis persuasive, we complemented it by testing the effect of several different linear motifs on circadian rhythmicity and in line with our bioinformatics screen, linear motifs that were enriched within circadian transcripts appeared to be implicated in circadian rhythmicity, among them the PXDLS motif.

Several lines of evidence support the involvement of the PXDLS motif in circadian rhythmicity through regulation of protein interactions within the core clock circuitry. First, expression of the PXDLS peptide disrupted circadian oscillations in a cell-autonomous manner, conceivably through interaction with the core clock proteins BMAL1/CLOCK and possibly due to the disruption of BMAL1/CLOCK binding to CRY1. Second, we discovered that BMAL1/CLOCK bind REV-ERBα, and that the evolutionary conserved PXDLS motif of REV-ERBα participates in the binding of REV-ERBα to BMAL1/CLOCK. Notably, BMAL1 have been previously reported to interact with CBP (34), which also harbors a conserved PXDLS motif, further supporting the possibility that BMAL/CLOCK have the capacity to bind PXDLS-containing proteins. Finally, we showed that the conserved PXDLS motifs of both NRIP1 and CBP play a role in circadian rhythmicity.

The nuclear receptor REV-ERBα has emerged as a bone fide core clock component within the molecular core clock machinery (6–9). To the best of our knowledge, our study is the first to report that BMAL1/CLOCK interact with REV-ERBα. Reciprocal co-immunoprecipitation experiments with endogenous proteins from mouse liver nuclear extracts evinced that BMAL1/CLOCK are in complex with REV-ERBα. Additional experiments in cultured cells further confirmed this interaction and suggested that it is PXDLS-dependent as a mutant form of REV-ERBα in its PXDLS motif exhibited reduced binding to BMAL1. Finally, the interaction of BMAL1 with REV-ERBα could be recapitulated using recombinant BMAL1 and *in vitro* translated [35S] labeled REV-ERBα. In conclusion, it appears that BMAL1/CLOCK and REV-ERBα can bind each other both *in vivo* and *in vitro*, yet we cannot conclusively determine whether the interaction is direct or mediated through auxiliary proteins that might be present in the purified complexes.

Our finding that BMAL1/ CLOCK interact with REV-ERBα and that the PXDLS motif is implicated in this interaction suggest an additional cross talk between the two established feedback loops, namely BMAL1/CLOCK-*Per/Cry* and REV-ERB/ROR-*Bmal1/Clock.* At this conjuncture, REV-ERBα has been recently reported to share hundreds of cistromic DNA binding regions with BMAL1 (7). Hence, it is conceivable that BMAL1/REV-ERBα complexes are transcriptionally functional and might function in concert to drive circadian gene expression of core clock and/or clock output genes. However, in contrast to the PXDLS motifs of NRIP1 and CBP, the PXDLS motif of REV-ERBα did not appear to play a role in the circadian rhythmicity of the core clock machinery as monitored with the *Bmal1*-luciferase reporter. It is possible that due to the strong repressor activity of REV-ERBα and the nature of the assay we used, in which we tested the effect of over expressed REV-ERBα, the functional differences between the wild type and mutant PXDLS could not be detected. Alternatively, it is possible that the PXDLS motif of REV-ERBα and the interaction of REV-ERBα with BMAL1/CLOCK do not play a role in circadian rhythmicity of the core clock oscillator but participate in regulation of clock output genes. Further studies are required to address this intriguing possibility.

Recent studies have focused on clock-modifying mechanisms/molecules (39–42). In particular, small molecule modifiers are emerging as powerful tools for understanding basic clock biology as well as developing putative therapeutic agents for clock-associated diseases. In this conjuncture, we show that short peptides (e.g. PXDLS) could be used to modulate circadian rhythmicity and thus might have potential therapeutic applications in the future.

## SUPPLEMENTARY DATA

Supplementary Data are available at NAR online.

SUPPLEMENTARY DATA
