# The PXDLS linear motif regulates circadian rhythmicity through protein–protein interactions

**DOI:** 10.1093/nar/gky629

**Published:** 2018-07-03

**Authors:** Moran Shalev, Rona Aviram, Yaarit Adamovich, Judith Kraut-Cohen, Tal Shamia, Shifra Ben-Dor, Marina Golik, Gad Asher

**Affiliations:** 1 Department of Biological Chemistry, Weizmann Institute of Science, Rehovot 76100, Israel; 2 Biological Services, Weizmann Institute of Science, Rehovot 76100, Israel

*Nucleic Acids Research, 2014*, https://doi.org/10.1093/nar/gku873

The authors wish to correct two figures, namely Figure 6D and Supplementary Figure S9 of the above-mentioned article.

**Figure 6.**
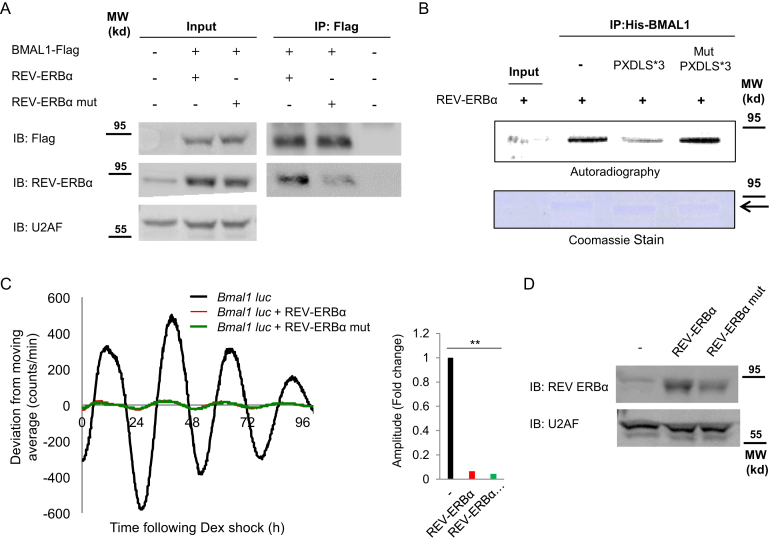
The PXDLS motif plays a role in BMAL1*/*CLOCK–REV-ERBα interaction. (**A**) NIH3T3 cells were transfected with BMAL1-Flag either with wild-type REV-ERBα or with PXDLS mutant REV-ERBα (REV-ERBα P72A) or non-transfected; protein lysates were prepared and immunoprecipitated with Flag antibody. Immunoprecipitated proteins were analyzed by SDS-PAGE and IB. (**B**) Pull down assay with recombinant His-BMAL1 coupled to beads and *in vitro* translated [35S] labeled REV-ERBα in the presence or absence of recombinant GST-PXDLS*3 or GST-Mut PXDLS*3 peptide. Recombinant BMAL1 was detected by Coomassie stained SDS-PAGE and [35S] labeled REV-ERBα by autoradiography. (**C**) NIH3T3 cells were transiently transfected with *Bmal1*-luciferase reporter either with control empty vector (black) or with wild-type REV-ERBα (red) or PXDLS mutant REV-ERBα (green). Cells were synchronized with a short dexamethasone (Dex) treatment, and real-time bioluminescence recording was performed using the LumiCycle. Amplitudes were quantified using the LumiCycle software. Data are presented on a bar graph as fold change (mean +*/*− SD, *n* = 4), *P*-values *<*0.001 is marked with **. (**D**) The expression levels of wild-type REV-ERBα and PXDLS mutant REV-ERBα were determined by SDS-PAGE and IB. U2AF was used as loading control. Molecular Weight (MW). An arrow indicates the full length BMAL1.

In Figure 6, the panel D was unintentionally duplicated from part of panel A in the same figure.

Supplementary Figure S9 is a composite image. The grouping of images was not clearly shown in the figure or explained in the caption. Dividing spacers have been added between non-adjacent Western-Blot lanes.

Supplementary Figure S8B presents the full blots of Figure 5C. The following sentence has been added to the figure legend: ‘The IB:BMAL1 panel in **(B)** was first blotted with CRY1 antibody and therefore contains residual signal from the CRY1 antibody next to the 72kd size marker’.

These corrections do not affect the results or conclusion of the article.

## Supplementary Material

Supplementary Data

