# Supplementary material for: The PXDLS linear motif regulates circadian rhythmicity through protein–protein interactions
Source: Nucleic Acids Res. 2014 Sep 26;42(19):11879–90. doi: 10.1093/nar/gku873 (PMC4231743; doi:10.1093/nar/gku873)
Supplement: SUPPLEMENTARY DATA [file supp_42_19_11879__index.html]

The PXDLS linear motif regulates circadian rhythmicity through protein–protein interactions — The PXDLS linear motif regulates circadian rhythmicity through protein–protein interactions — SUPPLEMENTARY DATA 

# The PXDLS linear motif regulates circadian rhythmicity through protein–protein interactions

## SUPPLEMENTARY DATA

**Files in this Data Supplement:**

- SUPPLEMENTARY DATA
- SUPPLEMENTARY DATA
- SUPPLEMENTARY DATA
