# Supplementary material for: The PXDLS linear motif regulates circadian rhythmicity through protein–protein interactions
Source: Nucleic Acids Res. 2014 Sep 26;42(19):11879–90. doi: 10.1093/nar/gku873 (PMC4231743; doi:10.1093/nar/gku873)
Supplement: SUPPLEMENTARY DATA [file supp_gku873_nar-02023-x-2014-File005.zip › NAR-02023-2014 Suppl files/Shalev_et_al.,_Supplementary_Table_S2_.docx]

| PXDLS*3 | Met D Y K D D D D K V L S T P S P S P L N L S S S R N S Q G Y T Y T A E G V Q E E P Q M E P L D L S L P K Q H G E L L E R S T I T S V Y Q N S V Y S V Q E E P L N L T C A K K E P S R |
| --- | --- |
| Mutant PXDLS*3 | Met D Y K D D D D K V L S T P S P S A S A S A S S R N S Q G Y T Y T A E G V Q E E P Q M E A S A S A L P K Q H G E L L E R S T I T S V Y Q N S L Y S V Q E E A S A S A C A K K E P S R |
| RGD*3 | Met D Y K D D D D K V L S T P S P S R G D N S Q G Y T Y T A E G V Q E E P Q M E R G D Q H G E L L E R S T I T S V Y Q N S V Y S V Q E E R G D K E P S R |
| PPXY*3 | Met D Y K D D D D K V L S T P S P S P P P Y N S Q G Y T Y T A E G V Q E E P Q M E P P P Y Q H G E L L E R S T I T S V Y Q N S V Y S V Q E E P P P Y K E P S R |
| TRAF*3 | Met D Y K D D D D K V L S T P S P S P E E S D E N S Q G Y T Y T A E G V Q E E P Q M E P E E S D E Q H G E L L E R S T I T S V Y Q N S V Y S V Q E E P E E S D E K E P S R |
| NPF*3 | Met D Y K D D D D K V L S T P S P S T N P F L N S Q G Y T Y T A E G V Q E E P Q M E T N P F L Q H G E L L E R S T I T S V Y Q N S V Y S V Q E E T N P F L K E P S R |
| PTAP*3 | Met D Y K D D D D K V L S T P S P S P T A P N S Q G Y T Y T A E G V Q E E P Q M E P T A P Q H G E L L E R S T I T S V Y Q N S V Y S V Q E E P T A P K E P S R |
| Synthetic PXDLS  (Flag spacers) | Met L E P L D L S L P K D Y K D D D D K D Y K D D D D K P L D L S L P K L E D Y K D D D D K D Y K D D D D K P L D L S L P K G S D Y K D D D D K D Y K D D D D K P L D L S L P K G S C A K K E P S R |
| Synthetic Mutant PXDLS  (Flag spacers) | Met L E A S A S A L P K D Y K D D D D K D Y K D D D D K A S A S A L P K L E D Y K D D D D K D Y K D D D D K A S A S A L P K G S D Y K D D D D K D Y K D D D D K A S A S A L P K G S C A K K E P S R |
