# Supplementary material for: The PXDLS linear motif regulates circadian rhythmicity through protein–protein interactions
Source: Nucleic Acids Res. 2018 Jul 3;46(14):7469–70. doi: 10.1093/nar/gky629 (PMC6101597; doi:10.1093/nar/gky629)
Supplement: Supplementary Data [file gky629_supplemental_files.zip › New_Figs_S8_S9.pptx]

## Slide 1
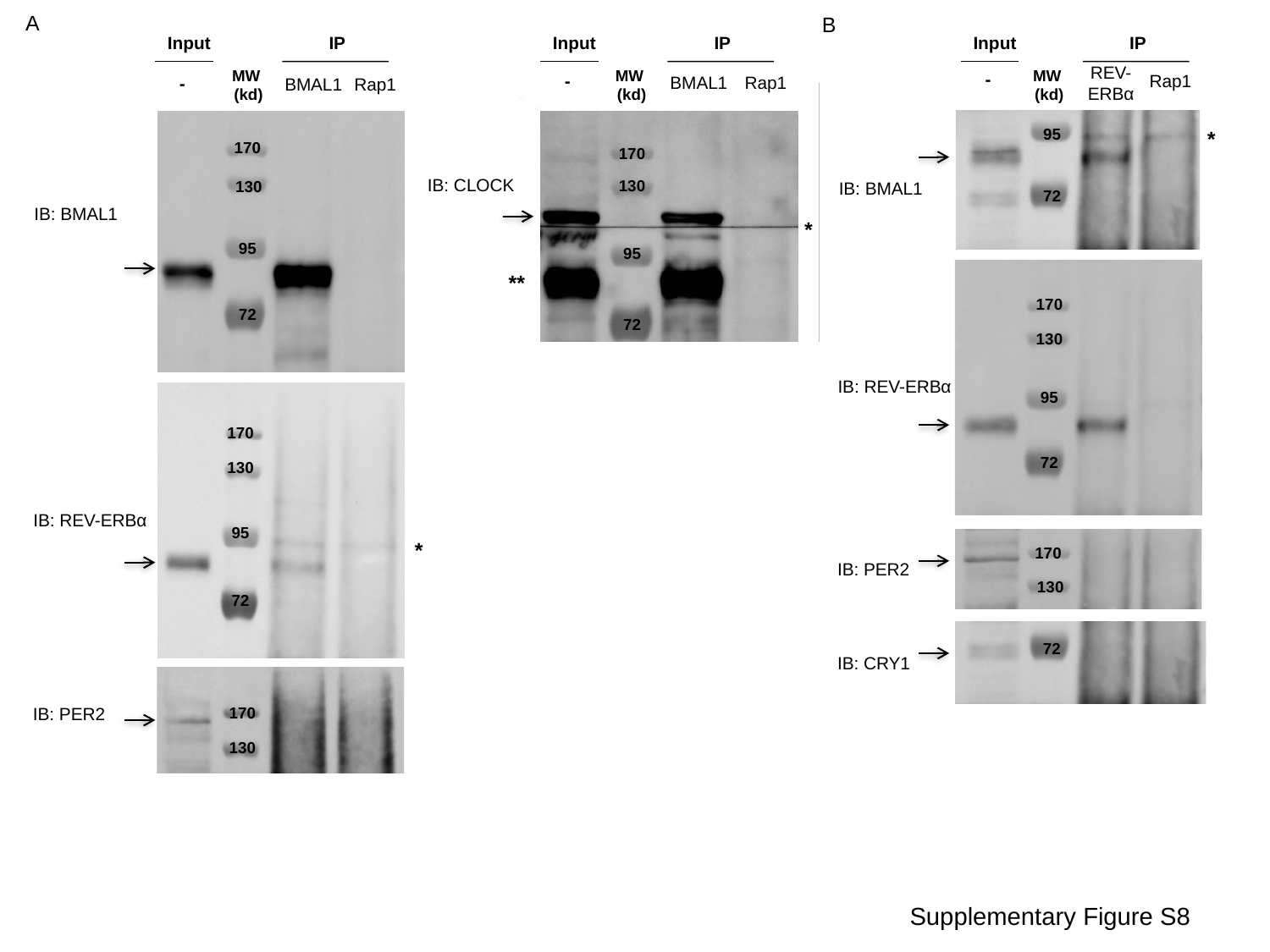

A
B
 Input
IP
 Input
IP
 Input
IP
REV-ERBα
MW
(kd)
MW
(kd)
MW
(kd)
-
 Rap1
-
 Rap1
BMAL1
-
 Rap1
BMAL1
95
72
*
170
130
95
72
170
130
95
72
IB: CLOCK
IB: BMAL1
IB: BMAL1
*
**
170
130
95
72
IB: REV-ERBα
170
130
95
72
IB: REV-ERBα
*
170
IB: PER2
130
72
IB: CRY1
IB: PER2
170
130
Supplementary Figure S8

## Slide 2
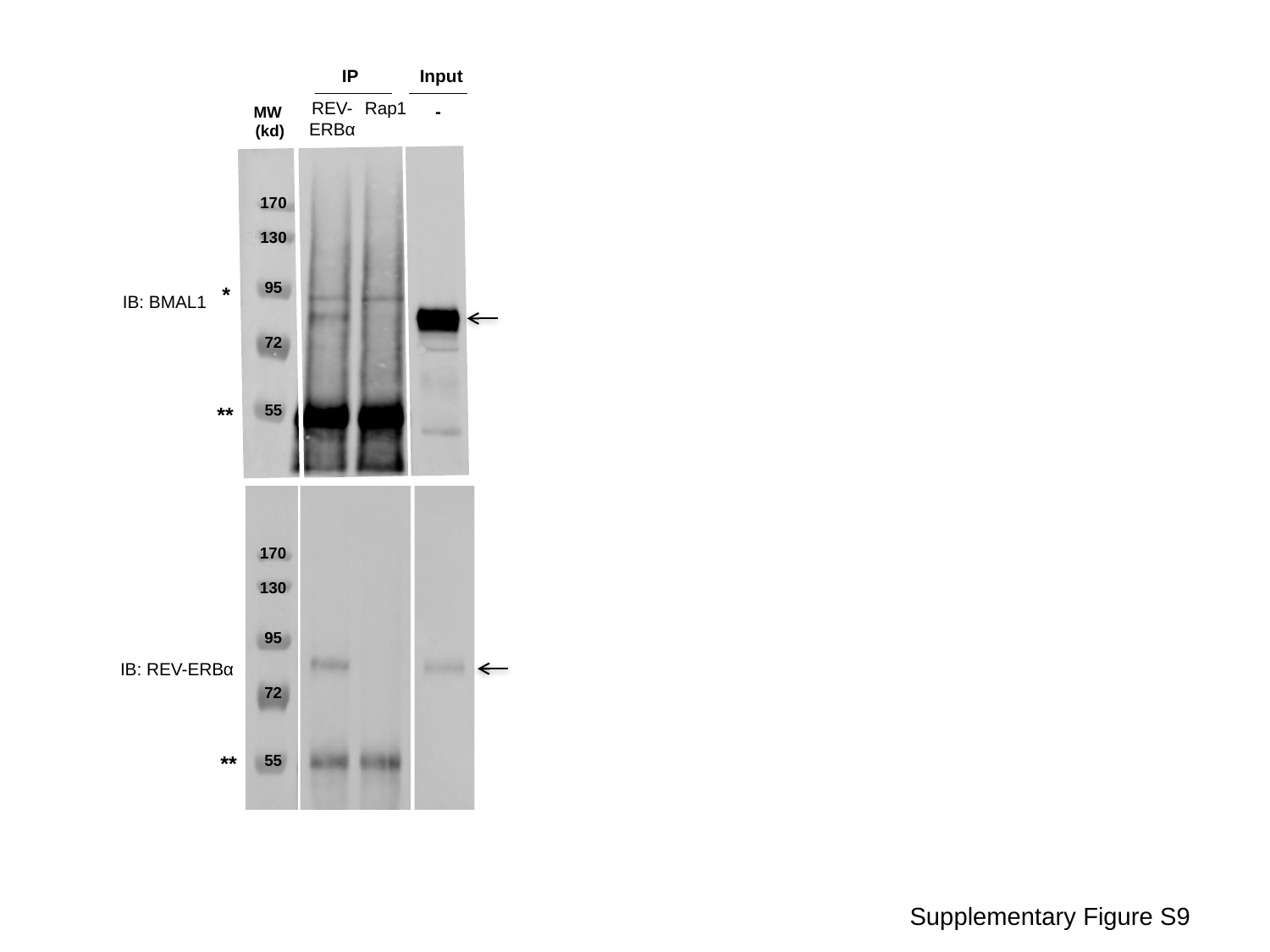

Input
IP
REV-ERBα
 Rap1
-
MW
(kd)
170
130
95
72
55
*
IB: BMAL1
**
170
130
95
72
55
IB: REV-ERBα
**
Supplementary Figure S9
