# Supplementary material for: The PXDLS linear motif regulates circadian rhythmicity through protein–protein interactions
Source: Nucleic Acids Res. 2018 Jul 3;46(14):7469–70. doi: 10.1093/nar/gky629 (PMC6101597; doi:10.1093/nar/gky629)
Supplement: Supplementary Data [file gky629_supplemental_files.zip › New_Supp_Captions.docx]

**Supplementary Figure S8. BMAL1/CLOCK interact with REV-ERB **Full blots of Figures 5B **(A)** and 5C **(B)**. The IB:BMAL1 panel in **(B)** was first blotted with CRY1 antibody and therefore contains residual signal from the CRY1 antibody next to the 72kd size marker. * - Non-specific band, ** - Residual signal from the BMAL1 immunoblotting. Molecular Weight (MW). Arrow indicates the protein of interest.

**Supplementary Figure S9. BMAL1/CLOCK interact with REV-ERBα.** A repetition of the experiment described in Figures 5C. * - Non-specific band, ** - Immunoglobulin heavy chain. Molecular Weight (MW). Arrow indicates the protein of interest.
