# Supplementary material for: The PXDLS linear motif regulates circadian rhythmicity through protein–protein interactions
Source: Nucleic Acids Res. 2018 Jul 3;46(14):7469–70. doi: 10.1093/nar/gky629 (PMC6101597; doi:10.1093/nar/gky629)
Supplement: Supplementary Data [file gky629_supplemental_files.zip › Supp_Figs.pptx]

## Slide 1
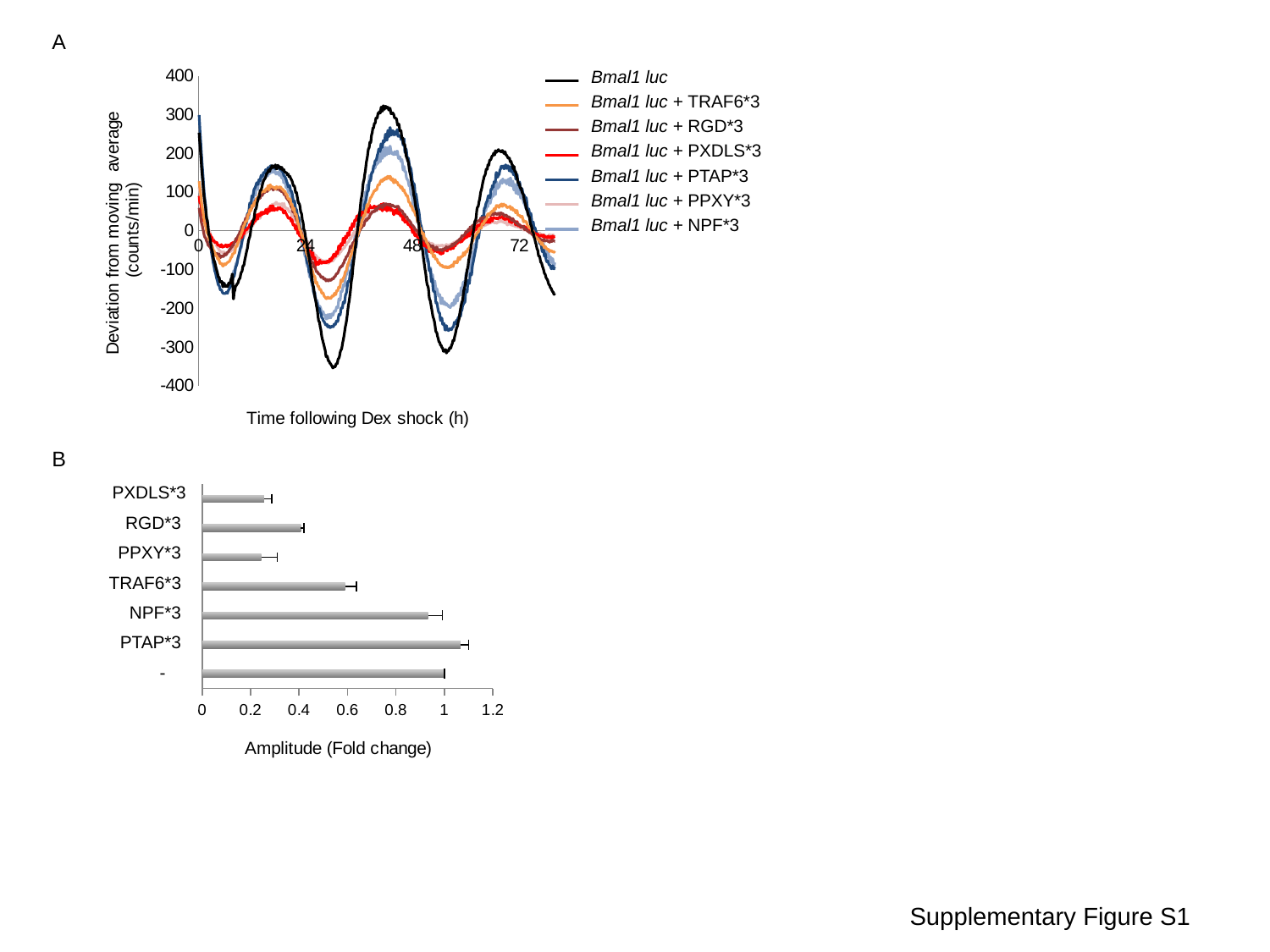

A
### Chart
| Category | Bmal luc | TRAF6 | RGD | PXDLS | PTAP | PPXY | NPF |
|---|---|---|---|---|---|---|---|Bmal1 luc
Bmal1 luc + TRAF6*3
Bmal1 luc + RGD*3
Bmal1 luc + PXDLS*3
Bmal1 luc + PTAP*3
Bmal1 luc + PPXY*3
Bmal1 luc + NPF*3
B
### Chart
| Category | |
|---|---|
| bmal luc | 1.0 |
| PTAP motif | 1.066666666666667 |
| NPF motif | 0.933333333333333 |
| TRAF6 motif | 0.590476190476191 |
| PPXY motif | 0.243809523809524 |
| RGD motif | 0.407619047619048 |
| PXDLS motif | 0.255238095238095 |PXDLS*3
 RGD*3
PPXY*3
TRAF6*3
NPF*3
PTAP*3
 -
Supplementary Figure S1

## Slide 2
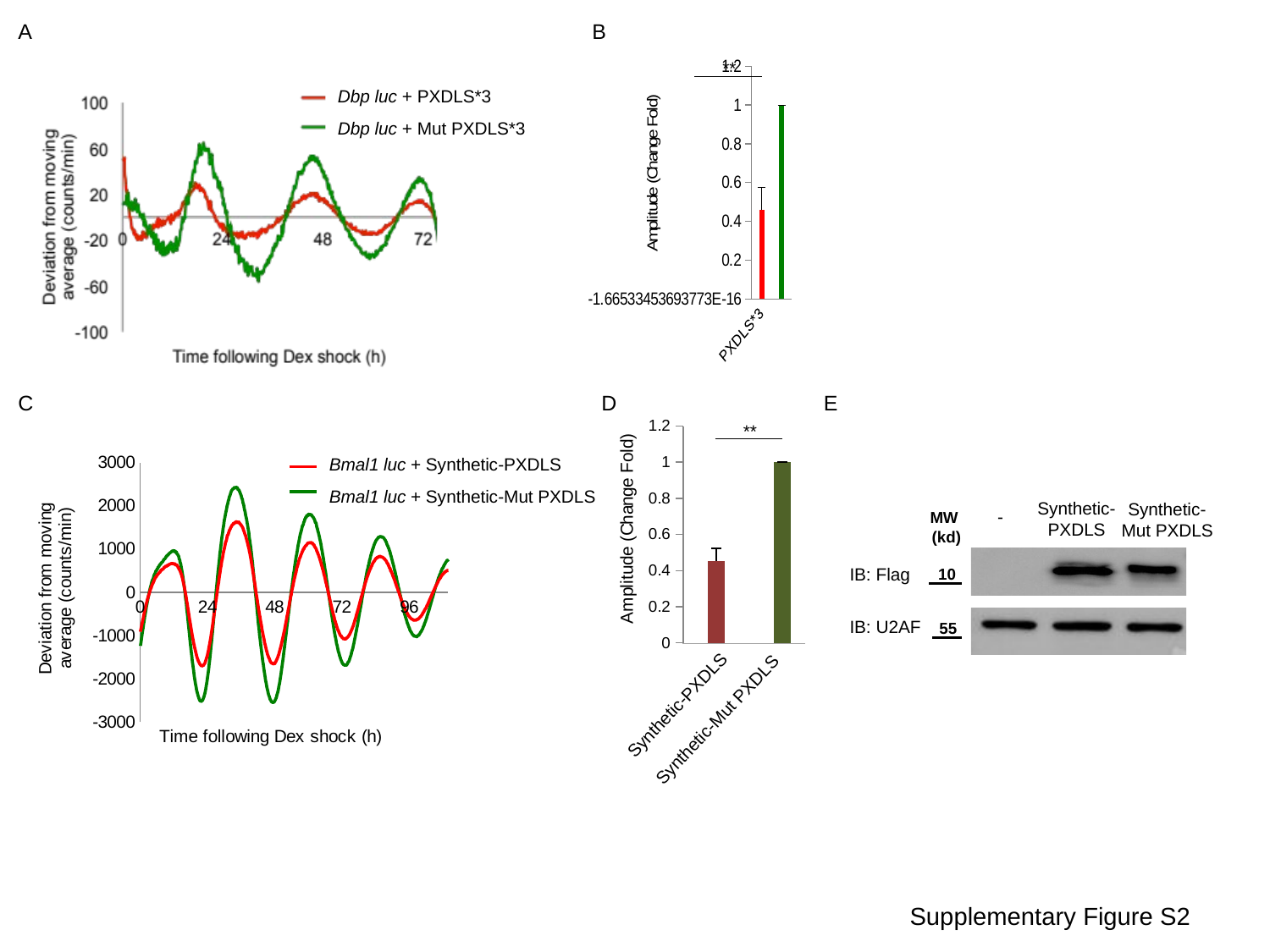

A
B
### Chart
| Category | |
|---|---|
| PXDLS*3 | 0.46 |
| Mut PXDLS*3 | 1.0 |**
Dbp luc + PXDLS*3
Dbp luc + Mut PXDLS*3
C
D
E
### Chart
| Category | |
|---|---|
| PXDLS*3 synthetic | 0.454177897574124 |
| mut PXDLS*3 synthetic | 1.0 |**
Bmal1 luc + Synthetic-PXDLS
Bmal1 luc + Synthetic-Mut PXDLS
### Chart
| Category | PLDLS synthetic | ASASA synthetic |
|---|---|---|Synthetic-
PXDLS
Synthetic-
Mut PXDLS
-
MW
(kd)
IB: Flag
10
IB: U2AF
55
Synthetic-PXDLS
Synthetic-Mut PXDLS
Supplementary Figure S2

## Slide 3
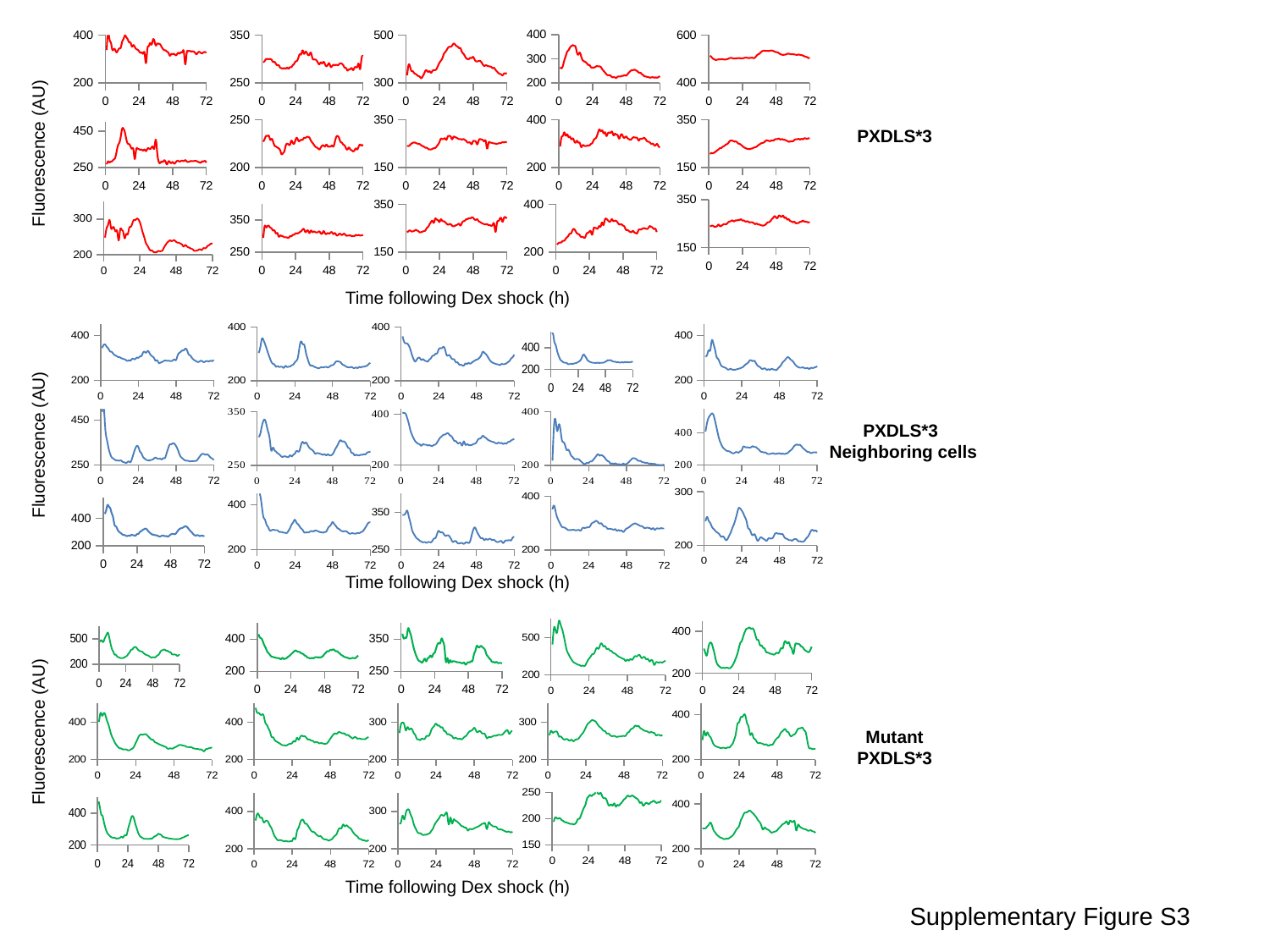

### Chart
| Category | |
|---|---|
### Chart
| Category | cell6 |
|---|---|
### Chart
| Category | cell23 |
|---|---|
### Chart
| Category | cell4 |
|---|---|
### Chart
| Category | cell6 |
|---|---|
### Chart
| Category | cell2 |
|---|---|
### Chart
| Category | cell14 |
|---|---|
### Chart
| Category | cell1 |
|---|---|
### Chart
| Category | cell18 |
|---|---|
### Chart
| Category | cell7 |
|---|---|PXDLS*3
Fluorescence (AU)
### Chart
| Category | cell10 |
|---|---|
### Chart
| Category | |
|---|---|
### Chart
| Category | cell10 |
|---|---|
### Chart
| Category | cell4 |
|---|---|
### Chart
| Category | cell14 |
|---|---|Time following Dex shock (h)
### Chart
| Category | cell44 |
|---|---|
### Chart
| Category | cell12 |
|---|---|
### Chart
| Category | cell22 |
|---|---|
### Chart
| Category | cell14 |
|---|---|
### Chart
| Category | cell33 |
|---|---|
### Chart
| Category | cell26 |
|---|---|
### Chart
| Category | cell47 |
|---|---|
### Chart
| Category | cell16 |
|---|---|
### Chart
| Category | cell1 |
|---|---|
### Chart
| Category | cell1 |
|---|---|PXDLS*3
Neighboring cells
Fluorescence (AU)
### Chart
| Category | cell8 |
|---|---|
### Chart
| Category | cell4 |
|---|---|
### Chart
| Category | cell32 |
|---|---|
### Chart
| Category | cell29 |
|---|---|
### Chart
| Category | cell15 |
|---|---|Time following Dex shock (h)
### Chart
| Category | CELL 2 |
|---|---|
### Chart
| Category | CELL 15 |
|---|---|
### Chart
| Category | CELL 14 |
|---|---|
### Chart
| Category | CELL 1 |
|---|---|
### Chart
| Category | cell6 |
|---|---|
### Chart
| Category | CELL 22 |
|---|---|
### Chart
| Category | CELL 11 |
|---|---|
### Chart
| Category | cell6 |
|---|---|
### Chart
| Category | cell11b |
|---|---|
### Chart
| Category | cell8 |
|---|---|Fluorescence (AU)
Mutant PXDLS*3
### Chart
| Category | |
|---|---|
### Chart
| Category | CELL 19 |
|---|---|
### Chart
| Category | CELL 23 |
|---|---|
### Chart
| Category | cell3 |
|---|---|
### Chart
| Category | cell10 |
|---|---|Time following Dex shock (h)
Supplementary Figure S3

## Slide 4
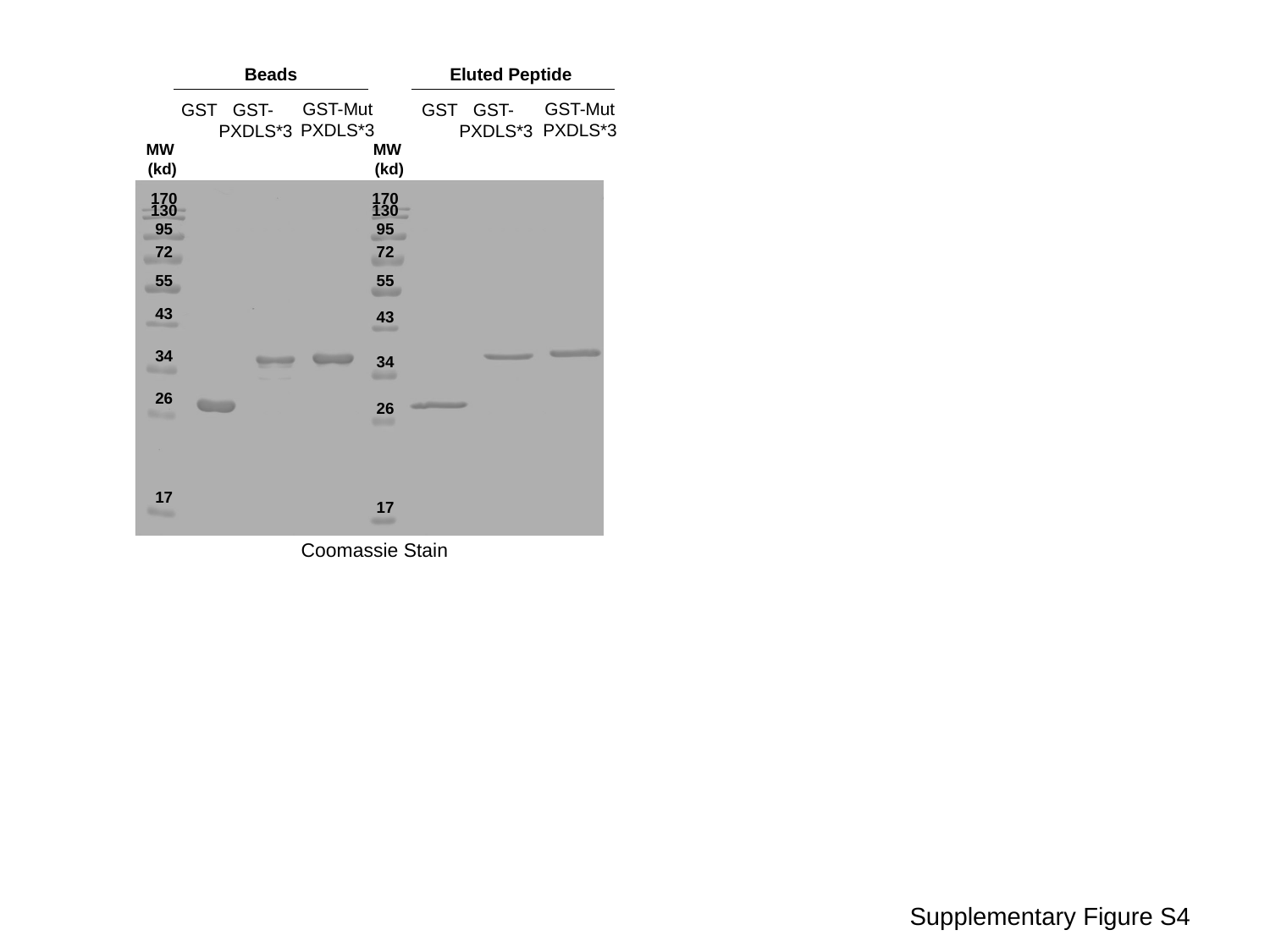

Beads
Eluted Peptide
GST-Mut PXDLS*3
GST-Mut PXDLS*3
GST
GST-
PXDLS*3
GST
GST-
PXDLS*3
MW
(kd)
MW
(kd)
170
130
95
72
55
43
34
26
17
170
130
95
72
55
43
34
26
17
Coomassie Stain
Supplementary Figure S4

## Slide 5
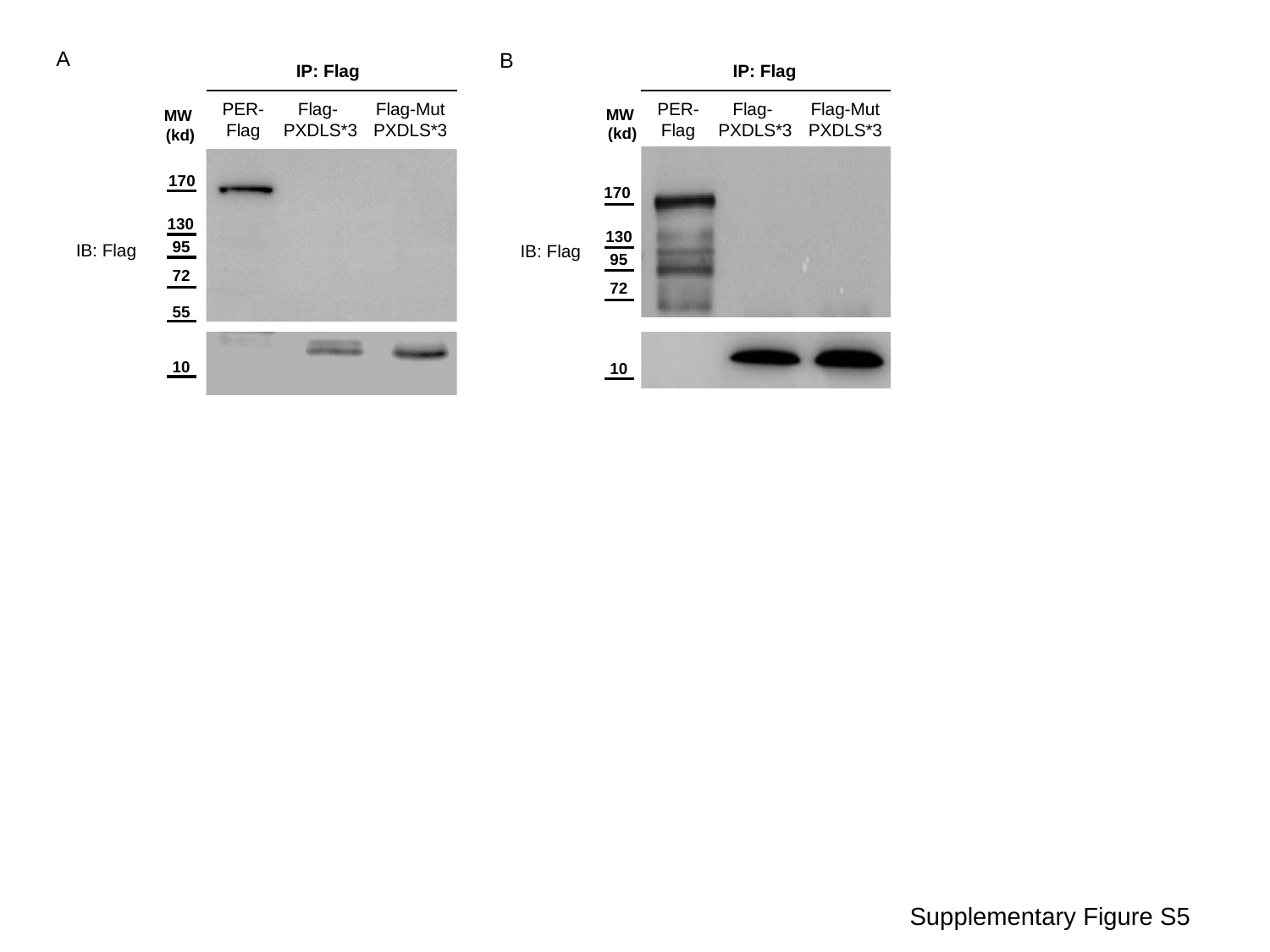

A
B
IP: Flag
IP: Flag
PER-Flag
Flag-
PXDLS*3
Flag-Mut PXDLS*3
PER-Flag
Flag-
PXDLS*3
Flag-Mut PXDLS*3
MW
(kd)
MW
(kd)
170
170
130
130
95
IB: Flag
IB: Flag
95
72
72
55
10
10
Supplementary Figure S5

## Slide 6
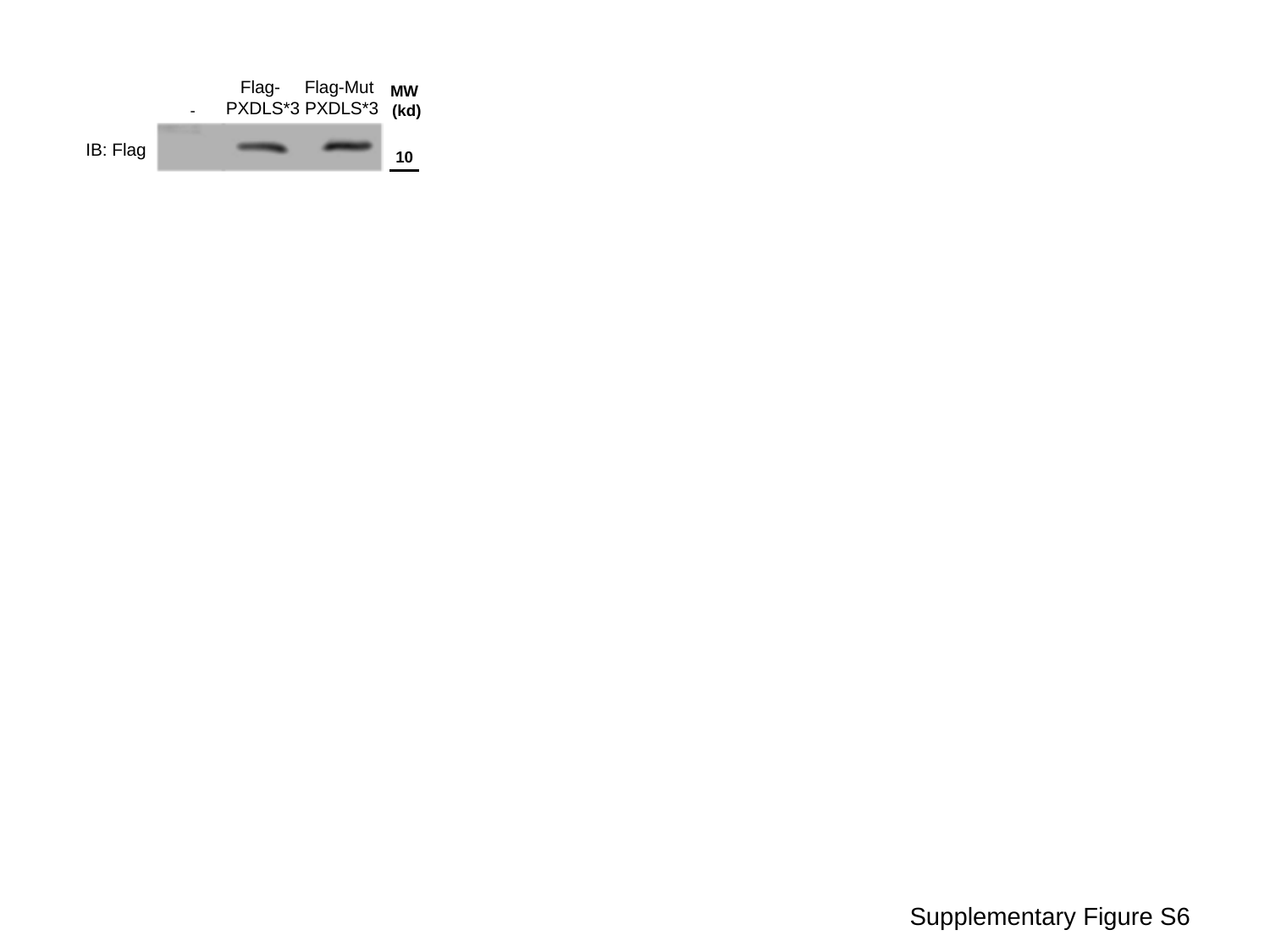

Flag-
PXDLS*3
Flag-Mut
PXDLS*3
MW
(kd)
-
IB: Flag
10
Supplementary Figure S6

## Slide 7
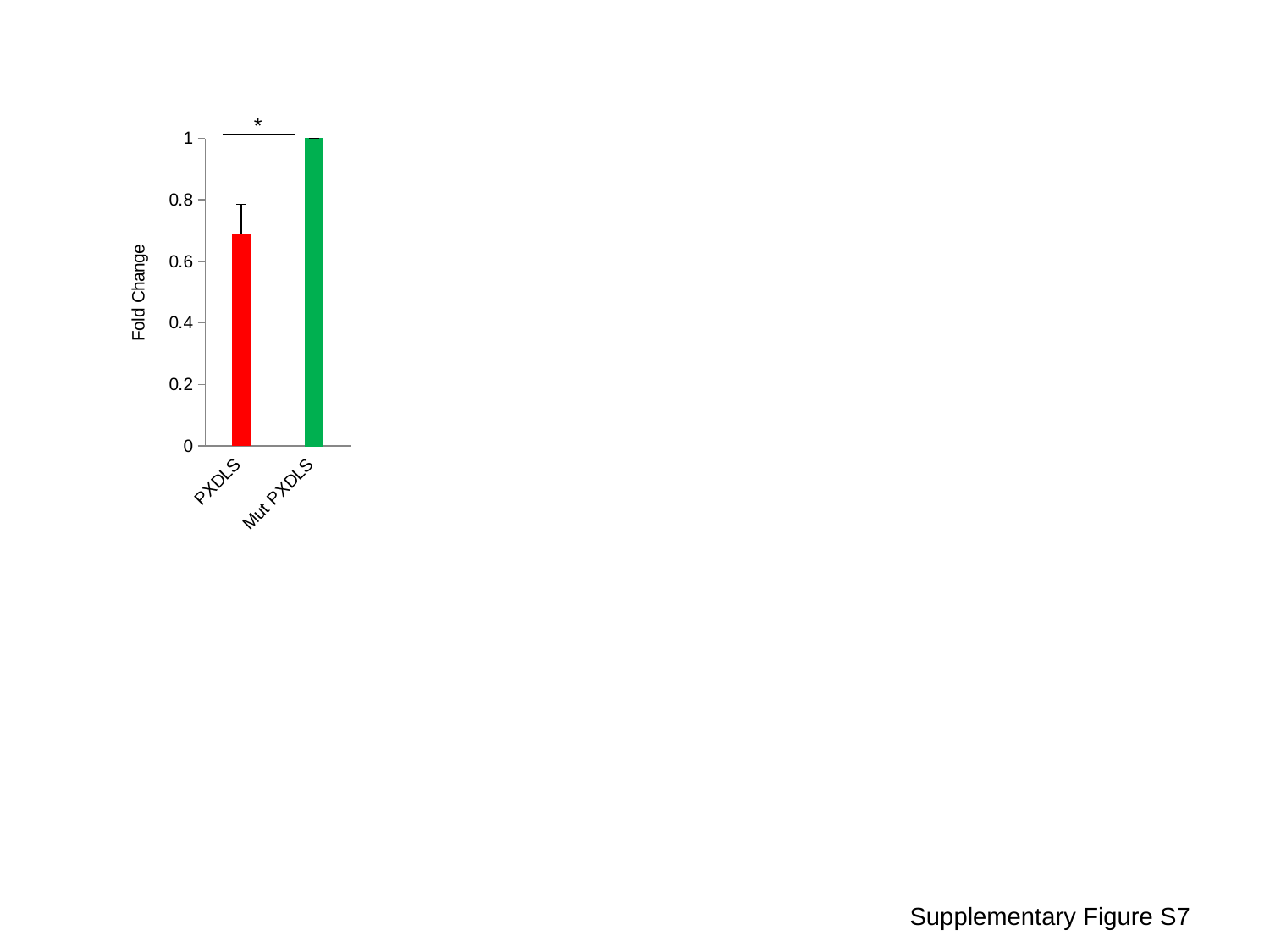

*
### Chart
| Category | |
|---|---|
| PXDLS | 0.690642036127211 |
| Mut PXDLS | 1.0 |Supplementary Figure S7

## Slide 8
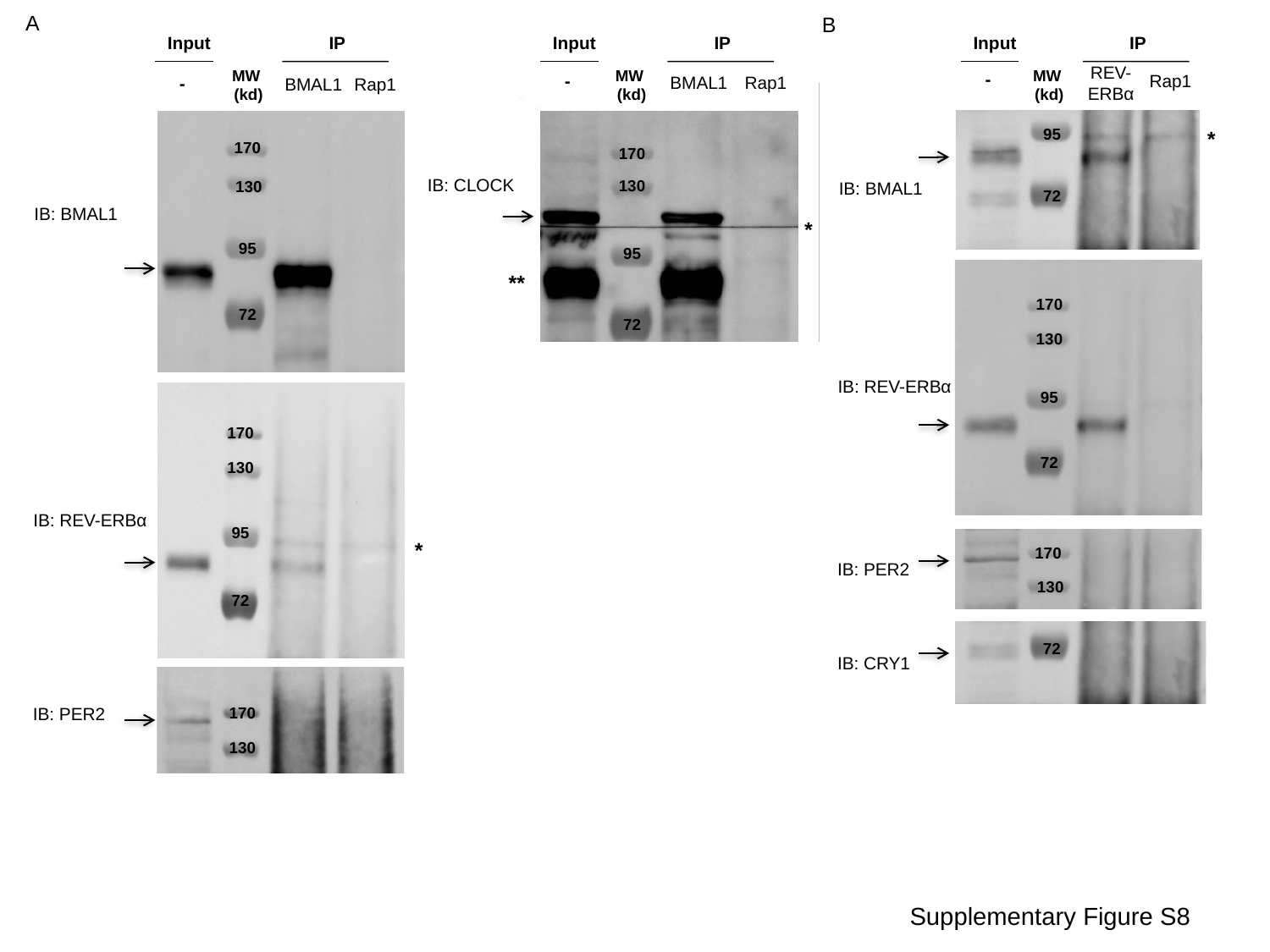

A
B
 Input
IP
 Input
IP
 Input
IP
REV-ERBα
MW
(kd)
MW
(kd)
MW
(kd)
-
 Rap1
-
 Rap1
BMAL1
-
 Rap1
BMAL1
95
72
*
170
130
95
72
170
130
95
72
IB: CLOCK
IB: BMAL1
IB: BMAL1
*
**
170
130
95
72
IB: REV-ERBα
170
130
95
72
IB: REV-ERBα
*
170
IB: PER2
130
72
IB: CRY1
IB: PER2
170
130
Supplementary Figure S8

## Slide 9
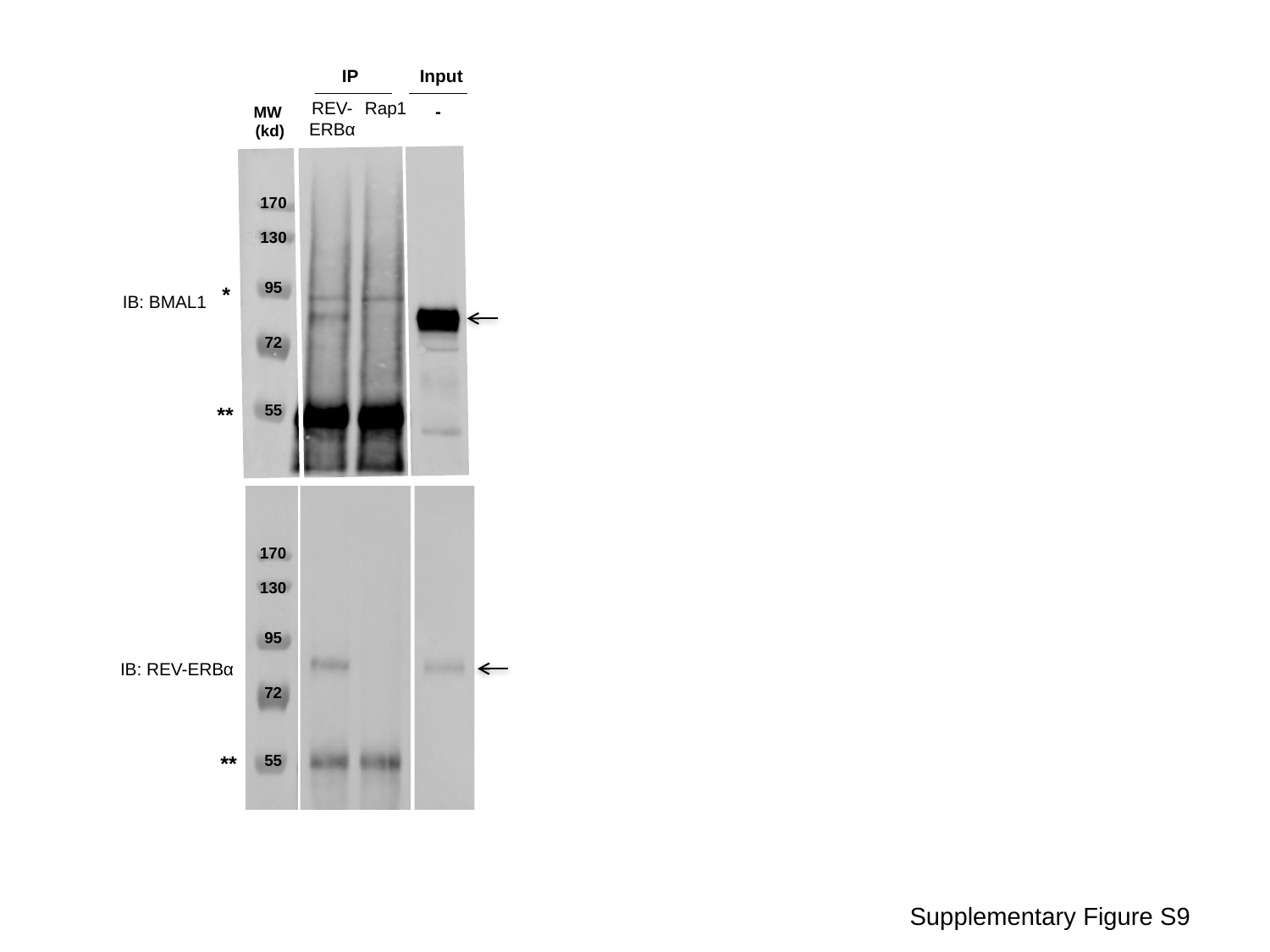

Input
IP
REV-ERBα
 Rap1
-
MW
(kd)
170
130
95
72
55
*
IB: BMAL1
**
170
130
95
72
55
IB: REV-ERBα
**
Supplementary Figure S9

## Slide 10
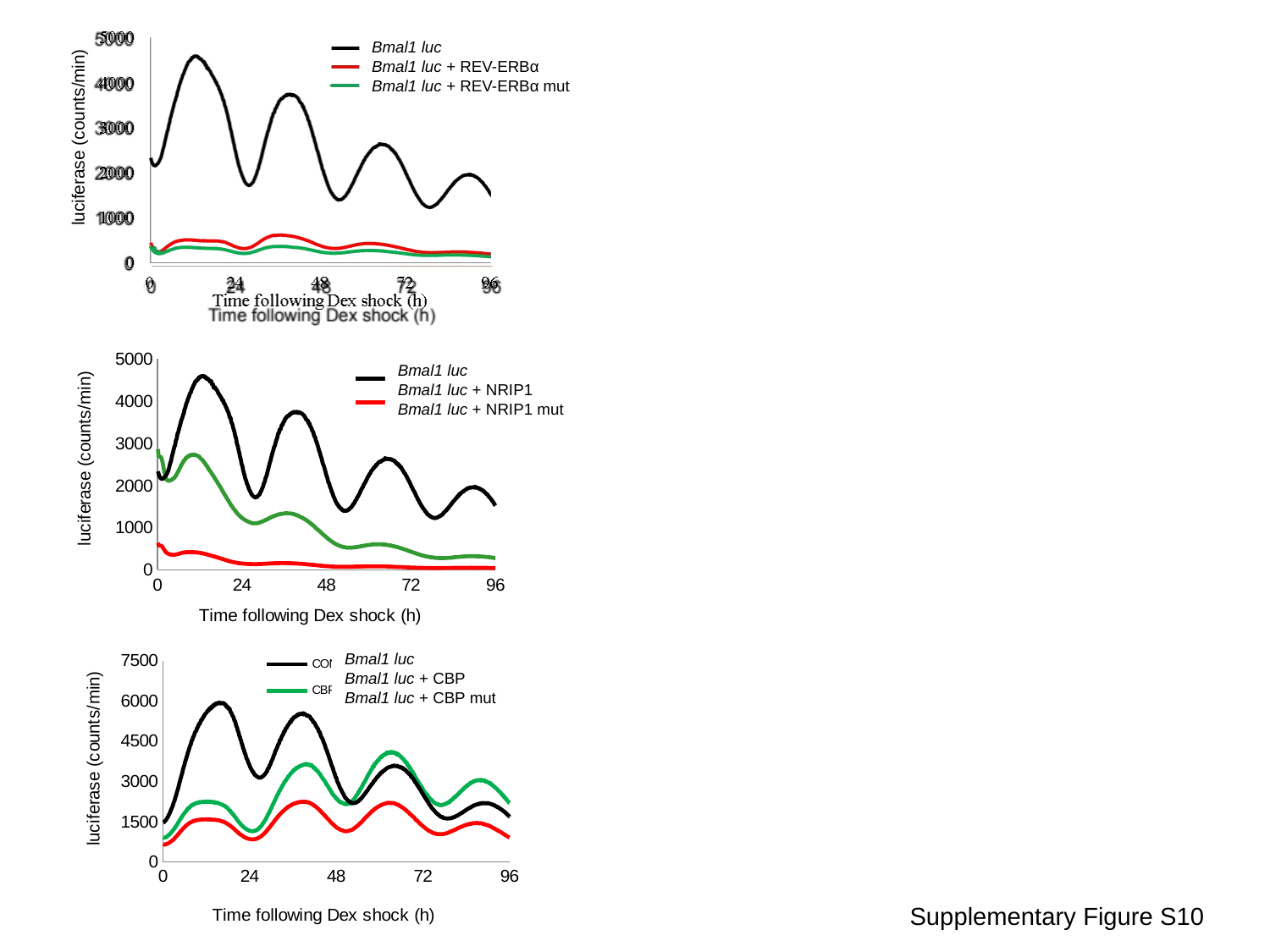

Bmal1 luc
Bmal1 luc + REV-ERBα
Bmal1 luc + REV-ERBα mut
luciferase (counts/min)
### Chart
| Category | Bmal luc | NRIP1 | NRIP1 mut1 |
|---|---|---|---|Bmal1 luc
Bmal1 luc + NRIP1
Bmal1 luc + NRIP1 mut
Bmal1 luc
Bmal1 luc + CBP
Bmal1 luc + CBP mut
### Chart
| Category | CONTROL-Bmal luc #45 | CBP | CBP mut |
|---|---|---|---|Supplementary Figure S10

## Slide 11
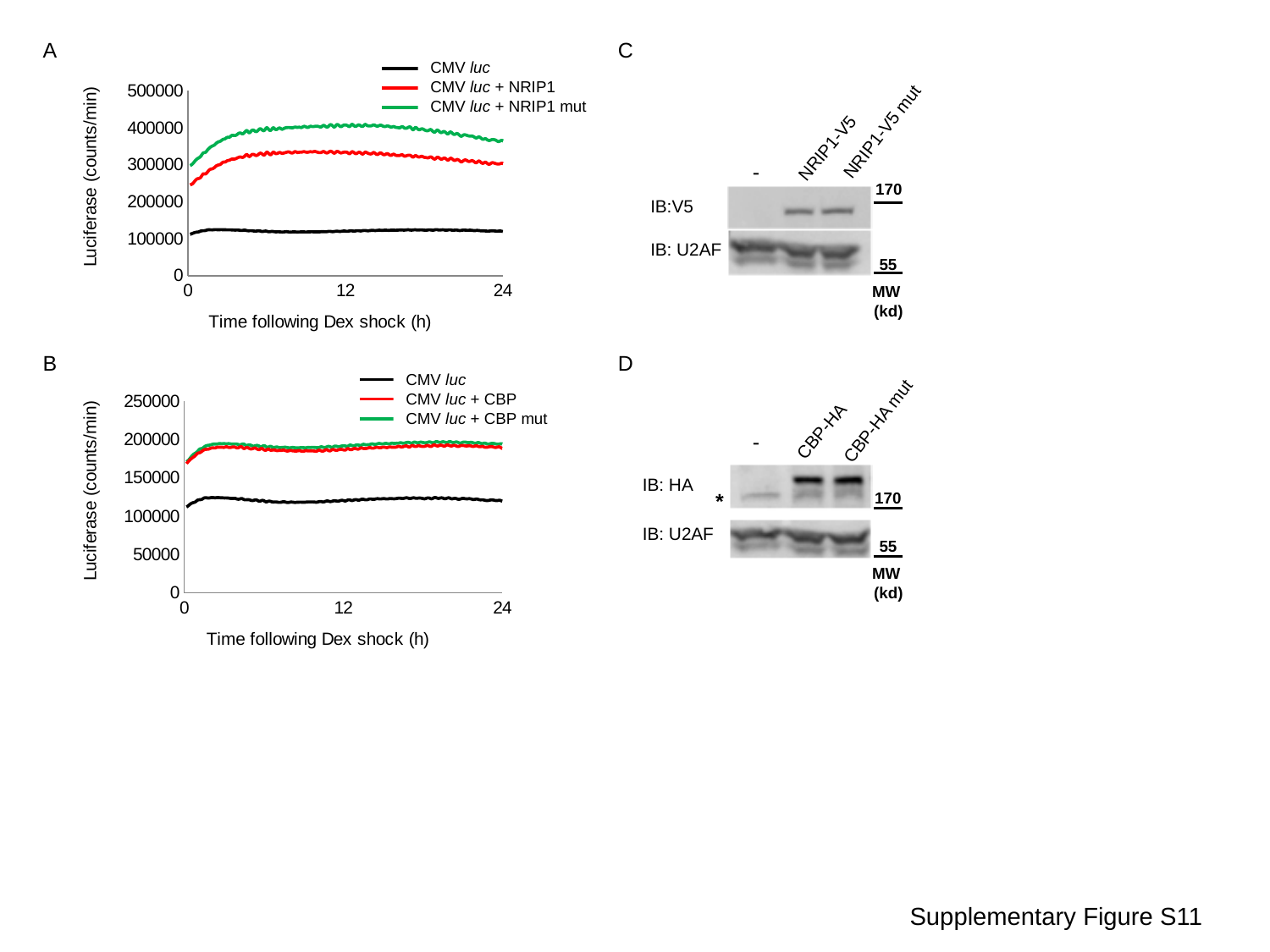

### Chart
| Category | cmv luc | NRIP1 | rip mut |
|---|---|---|---|A
C
CMV luc
CMV luc + NRIP1
CMV luc + NRIP1 mut
NRIP1-V5 mut
NRIP1-V5
-
170
| IB:V5 |
| --- |
| IB: U2AF |
55
MW
(kd)
### Chart
| Category | cmv luc | cbp | cbp mut old |
|---|---|---|---|B
D
CMV luc
CMV luc + CBP
CMV luc + CBP mut
CBP-HA mut
CBP-HA
-
| IB: HA |
| --- |
| IB: U2AF |
*
170
55
MW
(kd)
Supplementary Figure S11
