# Supplementary material for: The PXDLS linear motif regulates circadian rhythmicity through protein–protein interactions
Source: Nucleic Acids Res. 2018 Jul 3;46(14):7469–70. doi: 10.1093/nar/gky629 (PMC6101597; doi:10.1093/nar/gky629)
Supplement: Supplementary Data [file gky629_supplemental_files.zip › Supp_Text.docx]

**Supplementary Data**

**Supplementary Figure S1. The effect of different linear motifs on circadian rhythmicity.** (**A**) NIH3T3 cells were transiently transfected with *Bmal1*-luciferase reporter either alone, or together with transgene encoding 3 repeats in tandem of the following motifs TRAF6 (TRAF6*3), RGD (RGD*3), PXDLS (PXDLS*3), PTAP (PTAP*3), PPXY (PPXY*3) and NPF (NPF*3). Cells were synchronized with a short dexamethasone (Dex) treatment, and real-time bioluminescence recording were performed using the LumiCycle. (**B**) Amplitudes were quantified using the LumiCycle software. Data are presented on a bar graph as fold change (mean +/- SD, n=3).

**Supplementary Figure S2. The PXDLS motif disrupts circadian rhythmicity in a cell population**. (**A**) NIH3T3 cells were transiently transfected with *Dbp*-luciferase reporter either with the PXDLS*3 (Red) or the mutant PXDLS*3 (Green) transgene. Cells were synchronized with a short dexamethasone (Dex) treatment, and real-time bioluminescence recording were performed using the LumiCycle. (**B**) Amplitudes were quantified using the LumiCycle software. Data are presented on a bar graph as fold change (mean +/- SD, n=4). (**C**) NIH3T3 cells were transiently transfected with *Bmal1*-luciferase reporter either with PXDLS repeats with Flag epitope as spacers (Synthetic-PXDLS), (Red) or a mutant version (Synthetic-Mut PXDLS), (Green) transgene. (**D**) Amplitudes were quantified using the LumiCycle software. Data are presented on a bar graph as fold change (mean +/- SD, n=4). (**E**) The expression levels of the Synthetic-PXDLS and the Synthetic-Mut PXDLS were determined by SDS PAGE and immunoblot (IB). U2AF was used as loading control. Molecular Weight (MW), *P* values < 0.001 is marked in **.

**Supplementary Figure S3. The PXDLS motif disrupts circadian rhythmicity in single cells**. NIH3T3 cells expressing Venus-REV-ERBα reporter were transfected with Cherry expression vector either with the PXDLS*3 (Red) or the mutant PXDLS*3 transgene. Cells were synchronized with a short Dex treatment and single cells either expressing the Cherry fluorescent protein (i.e. PXDLS*3, or mutant PXDLS*3) or non-expressing cells (PXDLS*3 neighboring cells) were monitored and analyzed for their circadian oscillations using time-lapse fluorescence microscopy for three consecutive days at 1h resolution. Each plot represents fluorescence intensity profile for an individual cell. The single cell plots presented in this figure are additional to the ones presented in Figure 3. Arbitrary Units (AU).

**Supplementary Figure S4. Purification of GST, GST-PXDLS and GST-Mut PXDLS.** GST**,** GST-PXDLS and GST-Mut PXDLS were expressed in bacteria and affinity purified with glutathione agarose beads. GST, GST-PXDLS*3, and GST-Mut PXDLS*3 were eluted from the beads with 10mM reduced glutathione. The GST peptides coupled to glutathione beads and the eluted GST peptides were analyzed by Coomassie stained SDS-PAGE. Molecular Weight (MW).

**Supplementary Figure S5. BMAL1 and CLOCK bind to the PXDLS motif.** Immunoblots with Flag antibody of the immunoprecipitated proteins for the experiments described in Figure 4C **(A**) and Figure 4D **(B**). It should be noted that in view of the large size difference between the PER2-Flag and the PXDLS peptides, ~170kd and ~15kd respectively, the samples were analyzed on two separate and different SDS-PAGE and immunoblots. Molecular Weight (MW).

**Supplementary Figure S6. Purification of** **Flag-PXDLS*3 and Flag-Mut PXDLS*3 from HEK 293T cells.** Flag-PXDLS*3 or Flag-Mut PXDLS*3 transgenes were expressed in HEK 293T cells; protein lysates were prepared and immunoprecipitated with Flag antibody conjugated beads. The beads were extensively washed, subsequently Flag-PXDLS*3 and Flag-Mut PXDLS*3 were purified by elution with Flag peptide and analyzed by SDS PAGE and immunoblot (IB). Molecular Weight (MW).

**Supplementary Figure S7. The PXLDS peptide disrupts the binding of BMAL1 with CRY1.** Quantification of 3 individual experiments as described in Figure 4F, for the relative intensity of CRY1, upon immunoprecipitation of BMAL1 in the presence of GST-PXDLS*3 or GST-Mut PXDLS*3 peptides. Data are presented on a bar graph as fold change (mean +/- SD, n=3). *P* values < 0.05 is marked in *.

**Supplementary Figure S8. BMAL1/CLOCK interact with REV-ERB **Full blots of Figures 5B **(A)** and 5C **(B)**. The IB:BMAL1 panel in **(B)** was first blotted with CRY1 antibody and therefore contains residual signal from the CRY1 antibody next to the 72kd size marker. * - Non-specific band, ** - Residual signal from the BMAL1 immunoblotting. Molecular Weight (MW). Arrow indicates the protein of interest.

**Supplementary Figure S9. BMAL1/CLOCK interact with REV-ERBα.** A repetition of the experiment described in Figures 5C. * - Non-specific band, ** - Immunoglobulin heavy chain. Molecular Weight (MW). Arrow indicates the protein of interest.

**Supplementary Figure S10. The PXDLS-containing proteins, REV-ERB NRIP1, and CBP disrupt circadian oscillations of *Bmal1* luciferase reporter.** Representative unfiltered bioluminescence profiles of the experiments presented in Figures 6C, 7C, and 7D.

**Supplementary Figure S11. The effect of PXDLS-containing proteins, NRIP1 and CBP on CMV-luciferase reporter.** NIH3T3 cells were transiently transfected with CMV-luciferase reporter either with control empty vector (Black) or with (**A**) wild type NRIP1-V5 (Red) or PXDLS mutant NRIP1-V5 (Green), (**B**) wild type CBP-HA (Red) or PXDLS mutant CBP-HA (Green). Cells were synchronized with a short dexamethasone (Dex) treatment, and real-time bioluminescence recording were performed using the LumiCycle. The expression levels of (**C**) wild type NRIP1-V5 and PXDLS mutant NRIP1-V5, and (**D**) wild type CBP-HA and PXDLS mutant CBP-HA were determined by SDS PAGE and immunoblot (IB) with the indicated antibodies. U2AF was used as loading control. * - Non-specific band, Molecular Weight (MW).

**Supplementary Table S1. Transcripts list of PXDLS motif containing proteins.** A list of transcripts containing the different variant of the PXDLS motif, (i.e. PXDLSX(1,2)[KR], PXDLS, PX[NDS]LSX(1,2)[KR], PX[NDS]LS, PX[NDS]L[VTSAC]X(1,2)[KR]. Circadian transcripts are marked in bold.

**Supplementary Table S2. A sequence list of the transgenes used for expression of the different linear motifs.** A list detailing the transgenes used for the expression of the different linear motif and their peptide sequence. In each amino acid sequence the linear motif is highlighted in red.

**Supplementary Movie S1. The PXDLS motif disrupts circadian rhythmicity in single cells.** Representative movies of fluorescence recording of NIH3T3-Rev-VNP, co-transfected with Cherry expression vector either with (**A**) the PXDLS*3 or (**B**) the mutant PXDLS*3 transgene. Fluorescent signal was recorded using time-lapse fluorescence microscopy at 1 h resolution.
